# A Nascent Peptide Signal Responsive to Endogenous Levels of Polyamines Acts to Stimulate Regulatory Frameshifting on Antizyme mRNA*[Fn FN2]

**DOI:** 10.1074/jbc.M115.647065

**Published:** 2015-05-21

**Authors:** Martina M. Yordanova, Cheng Wu, Dmitry E. Andreev, Matthew S. Sachs, John F. Atkins

**Affiliations:** From the ‡ School of Biochemistry and Cell Biology, University College Cork, Cork, Ireland,; the § Department of Biology, Texas A&M University, College Station, Texas 77843-3258,; the ¶ Belozersky Institute of Physico-Chemical Biology, Lomonosov Moscow State University, Moscow 119991, Russia, and; the ‖ Department of Human Genetics, University of Utah, Salt Lake City, Utah 84112-5330

**Keywords:** mRNA, polyamine, ribosome, RNA structure, translation regulation, ORF, antizyme, frameshifting

## Abstract

The protein antizyme is a negative regulator of cellular polyamine concentrations from yeast to mammals. Synthesis of functional antizyme requires programmed +1 ribosomal frameshifting at the 3′ end of the first of two partially overlapping ORFs. The frameshift is the sensor and effector in an autoregulatory circuit. Except for *Saccharomyces cerevisiae* antizyme mRNA, the frameshift site alone only supports low levels of frameshifting. The high levels usually observed depend on the presence of *cis*-acting stimulatory elements located 5′ and 3′ of the frameshift site. Antizyme genes from different evolutionary branches have evolved different stimulatory elements. Prior and new multiple alignments of fungal antizyme mRNA sequences from the Agaricomycetes class of Basidiomycota show a distinct pattern of conservation 5′ of the frameshift site consistent with a function at the amino acid level. As shown here when tested in *Schizosaccharomyces pombe* and mammalian HEK293T cells, the 5′ part of this conserved sequence acts at the nascent peptide level to stimulate the frameshifting, without involving stalling detectable by toe-printing. However, the peptide is only part of the signal. The 3′ part of the stimulator functions largely independently and acts at least mostly at the nucleotide level. When polyamine levels were varied, the stimulatory effect was seen to be especially responsive in the endogenous polyamine concentration range, and this effect may be more general. A conserved RNA secondary structure 3′ of the frameshift site has weaker stimulatory and polyamine sensitizing effects on frameshifting.

## Teaser

**Background:** Regulatory +1 frameshifting in antizyme mRNA is central for polyamine level homeostasis.

**Results:** A nascent peptide stimulator of +1 frameshifting operates in Agaricomycete fungal antizyme mRNA.

**Conclusion:** Frameshifting can be stimulated by nascent peptide interactions with the ribosome's peptide exit tunnel in a polyamine-dependent manner.

**Significance:** Specific nascent peptide stimulators may be a more general and unappreciated feature of utilized ribosomal frameshifting.

## Introduction

Notwithstanding some early antibiotic studies, shortly after determination of the first structure of the “tube” through which the nascent peptide passes from the internal ribosome site of its synthesis to the ribosome's exterior, the interior of this exit tunnel was thought to behave like “molecular Teflon” and not interact with nascent peptide sequence, thereby allowing unimpeded peptide egress (1). However, interactions between certain specific amino acid sequences and tunnel components do occur, and evolution has exploited this for diverse and important functions.

These interactions can modulate translation downstream from sequence encoding the interacting segment of the nascent peptide (2). To what extent such consequences are influenced by ribosome conformational changes associated with the interactions and to what extent by stalling of nascent peptide progression are likely case-specific.

Nascent peptides encoded by eukaryotic regulatory upstream open reading frames (uORF)^2^ can induce ribosomal stalling at uORF termination codons, thus providing a physical barrier for scanning ribosomes with resultant inhibition of downstream main ORF translation. Some regulatory nascent peptides act in concert with small molecules, such as amino acids or polyamines. A highly conserved uORF within the leader sequence of the polyamine biosynthetic enzyme AdoMetDC mRNA from mammals encodes a peptide with the sequence MAGDIS. This, in response to an increase in polyamines levels, causes the ribosome to stall at the termination codon, with consequent inhibition of translation of the downstream main ORF (3). A uORF 5′ of the gene for a fungal arginine biosynthetic enzyme encodes an arginine attenuator peptide (AAP). In response to high arginine concentration, the nascent AAP causes ribosomes to stall at the uORF termination codon, thereby blocking ribosomes from translating the downstream main ORF (4).

Macrolides promote the expression of bacterial resistance genes by inducing ribosome stalling during synthesis of an upstream-encoded leader peptide (5). Interestingly, ketolides have been shown to promote −1 frameshifting in decoding the leader peptide without inducing detectable ribosome stalling (6).

Here we explore the possibility that a specific nascent peptide sequence acts as a stimulatory signal for ribosomal frameshifting utilized positively for gene expression. The work focuses on antizyme mRNA frameshifting.

**FIGURE 1.**
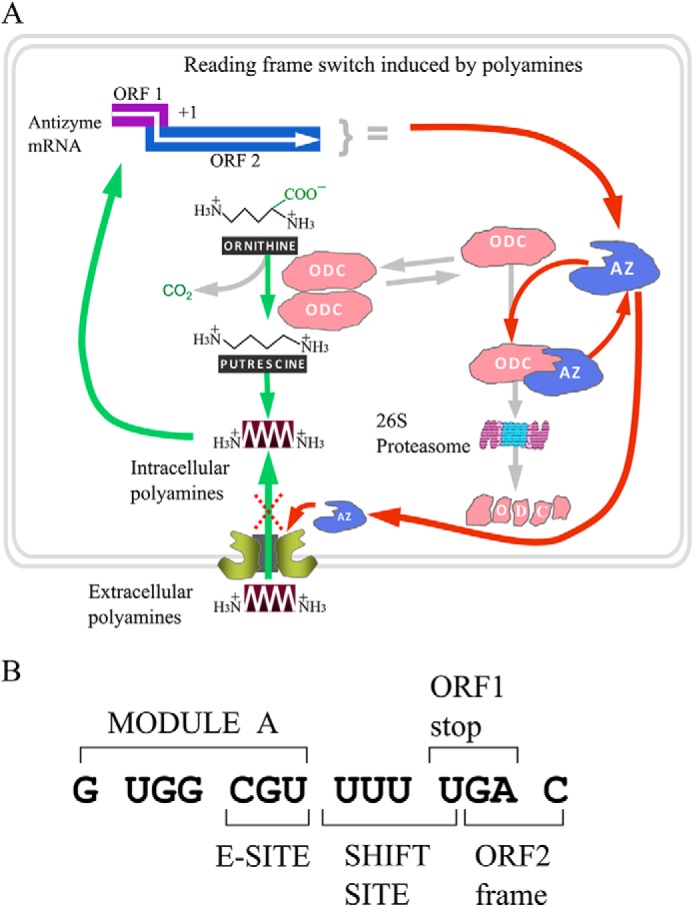
**Regulatory frameshifting in antizyme mRNA is a central event for maintaining polyamine homeostasis.** *A*, schematic representation of the role of the negative regulator, antizyme, in maintaining polyamine homeostasis. Ornithine decarboxylase (*ODC*) catalyzes the first and rate-limiting step in the polyamine synthesis. Antizyme disrupts the active ornithine decarboxylase homodimer and targets it for ubiquitin independent degradation by the 26S proteasome. Antizyme also regulates the polyamine membrane transporter by inhibiting polyamine uptake and stimulating polyamine excretion. Antizyme protein synthesis requires translational frameshifting, which is stimulated by free intracellular polyamines. This event completes an autoregulatory circuit. *B*, *C. cinerea* antizyme mRNA sequence in the vicinity of the frameshift site.

The protein antizyme occurs in cells from yeast to mammals. It targets specific proteins for ubiquitin-independent proteosomal-mediated degradation. Its best known interaction is with ornithine decarboxylase that catalyzes synthesis of putrescene, the precursor of the polyamines spermidine and spermine (7–9). In addition to inhibiting the synthesis of polyamines, antizyme also inhibits extra cellular uptake of polyamines (10) (Fig. 1*A*). Given the importance of the small organic polycations, polyamines, for a wide range of cellular processes (11–13), the major consequences of distortions of their level (14–17), and probably other roles of antizyme (18), it is not surprising that antizyme synthesis is itself highly regulated.

The level of antizyme is governed in several ways including sequestration by inhibitory proteins whose own synthesis is polyamine-regulated (19–21), as well as by C-Jun effects on polyamine catabolism (22). Antizyme is encoded by two partially overlapping reading frames, and a programmed +1 ribosomal frameshifting event at the end of the first ORF is required for synthesis of functional antizyme. (Fig. 1) (23). The frameshifting involved is a crucial sensor and effector of an autoregulatory circuit because elevated polyamines result in the synthesis of more antizyme, which dampens both the synthesis and uptake of polyamines, thereby restoring optimal polyamine levels. Frameshifting efficiency can also be responsive to other conditions. In *Saccharomyces cerevisiae*, the prion state of the translation termination factor eRF3, which is of evolutionary significance in stress conditions, enhances antizyme frameshifting (24). Nutritional stress increases antizyme expression in a polyamine-independent manner (25). Irrespective of this, the degree of the known effect of polyamines on the specific utilized antizyme frameshift event is much greater than the enhanced differential effect that polyamines can have on the synthesis of diverse proteins (26, 27).

Recoding signals serve to potentiate high levels of frameshifting at the relatively inefficient frameshift site at the end of ORF1 in most antizyme mRNAs. An exception is the budding yeast *S. cerevisiae* and presumably closely related species, in which the recoding signals serve to reduce, in a polyamine-dependent manner, the inherently very high level of frameshifting at the shift site in budding yeast (28). Effects of the nascent peptide within the exit tunnel of the ribosome in which it was just synthesized are an important component of the negatively acting recoding signals in *S. cerevisiae* antizyme frameshifting (28). However, two contrasting sets of results (28, 29) need reconciliation. Interestingly, studies in *S. cerevisiae* have shown that the relative concentrations of spermidine and putrescine are important for frameshifting efficiency (30).

In contrast to the nascent peptide effects in *S. cerevisiae*, all the experimentally investigated antizyme frameshifting signals from *Schizosaccharomyces pombe* to humans act at the RNA level (31). These recoding signals are both 5′ and 3′ of the shift site. Different mRNA pseudoknots are strongly represented in the 3′ signals (23, 32). As judged by deletion analyses, the 5′ stimulatory sequence in rats involves 50 nucleotides immediately upstream of the frameshift site. Although all are required for optimal levels of frameshifting, the sequence of the three codons just 5′ of the ORF1 stop codon have the greatest effect (23). Comparative sequence analysis has revealed that the 5′ stimulatory sequence has a modular structure with the different modules evolving independently in the different evolutionary clades. The closest module to the shift site, module A, is the most highly conserved (31). In contrast to the results from the deletion analysis, single nucleotide substitutions of what is now called module A of mammalian antizyme 1 showed only a modest effect on frameshift efficiency (23). Without further experimental testing, it was considered possible that it may act via interaction with rRNA of the mRNA exit tunnel, based on the precedent of 5′ stimulators for other cases of frameshifting being known to act in this manner (33, 34).

With the exception of one high level case of incidental bacterial frameshifting (35), all non-antizyme investigated viral and chromosomal cases of programmed frameshifting involve recoding signals that act at the RNA level. Nevertheless, prior phylogenetic analysis of one case of positively utilized frameshifting where stimulatory signals are required to boost frameshifting efficiency indicated a recoding signal that acts at the nascent peptide level. This analysis was of the antizyme mRNA sequence from the 10 species then known from the Agaricomycotina subphylum of Basidiomycota fungi. One of these species was *Coprinopsis cinerea*. A highly conserved region of about 40 nucleotides was identified, and its 3′ boundary is approximately at the 5′ boundary of module A and so 5′ of the frameshift site. The pattern of conservation in this region suggested that it functions at the peptide level and not at the nucleotide level (36).

**FIGURE 2.**
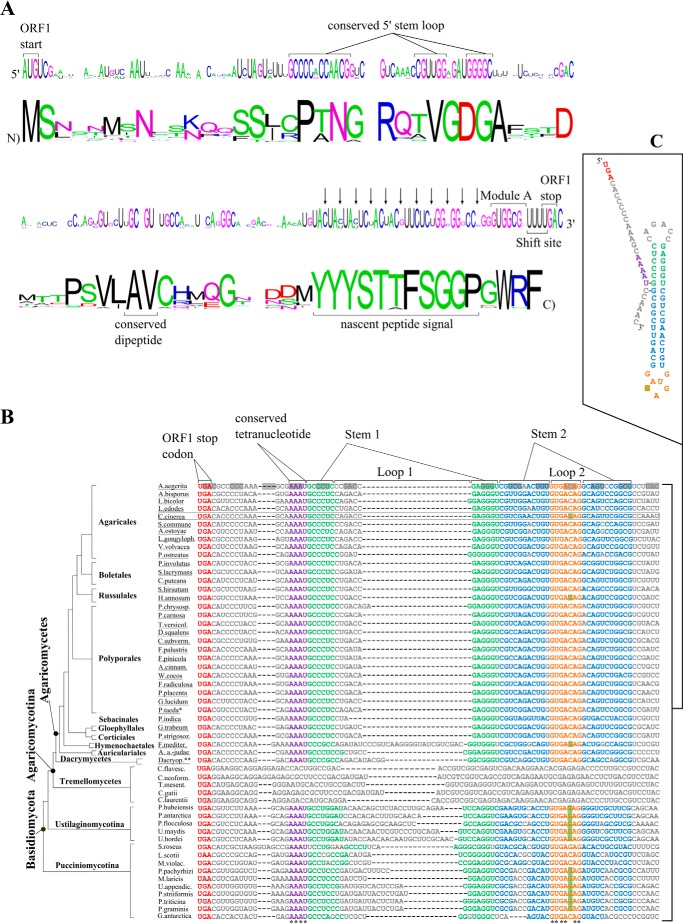
**Conservation of antizyme mRNA sequences from Basidiomycota, and their encoded products.** *A*, an alignment of 35 ORF1 antizyme mRNA sequences was used to generate the nucleotide and amino acid logos shown here. Of these sequences, 34 belong to Agaricomycetes, and 1 belongs to Dacrymycetes (supplemental Sequence List S1). Starting from the 3′ end of the nucleotide logo indicated are the ORF1 stop codon, the shift site, the sequence of module A, and the conserved peptide encoding region. Other conserved features more distal to the shift site, such as a conserved dipeptide and a conserved 5′ stem loop, are also indicated. The gaps in the logos indicate that each nucleotide or amino acid residue occurs with equal probability at the corresponding position. *B*, alignment of 54 Basidiomycotal antizyme mRNA sequences 3′ of the shift site. The region shown here contains the conserved 3′ RNA structure. The nucleotide variations in loop 2 are highlighted in *green*. The correct reading frame is indicated in the first sequence of the alignment. The absolutely conserved four nucleotide sequence abutting stem 1 are shown in *magenta*. Completely conserved nucleotides are indicated by an *asterisk* below the alignment. The four sequences of the Tremellomycetes class that lack the potential RNA structure were left uncolored and were not taken into account when analyzing the alignment. The *underlined names* are of the sequences used for the alignments in Fig. 2*A*. The scheme of the phylogenetic tree was adapted from the work of Floudas *et al.* (41) and is not intended to indicate precise evolutionary distances and scales. P.taeda* refers to an EST derived from a *Pinus taeda* library that is an Agaricomycetes contaminant of unknown identity. *Dacryop*.** stands for *Dacryopinax sp. C*, the predicted secondary structure of the putative RNA stimulator, inferred from the alignment, featuring the *C. cinerea* mRNA sequence; the *coloring* corresponds to that of the alignment.

The putative stimulatory element 3′ of the frameshift site is a potential RNA secondary structure starting 19 nucleotides downstream of the frameshift site. The structure consists of two directly adjacent stem loops, suggesting that they may co-axially stack on each other (Fig. 2, *B* and *C*). Its position relative to the frameshift site suggests that it would be just outside the ribosome mRNA tunnel during the frameshift event (36). With the exception of two recently described cases (37, 38), this is different from the other previously described stimulatory structures that are located closer to the frameshift site and presumed to act from within the mRNA entrance tunnel at the mRNA unwinding site (23).

Antizyme homologs are present in diverse eukaryotic evolutionary branches, including in animals, fungi, and protists (31, 36, 39, 40). However, there is no indication that antizyme is present in plants. Fungal antizymes sequences have been identified in six separate phyla: Ascomycota, Basidiomycota, Glomeromycota, Zygomycota, Neocallimastigomycota, and Blastocladiomycota (36).^3^ The Basidiomycota phylum contains three subphyla: Pucciniomycotina, Ustilaginomycotina, and Agaricomycotina. Agaricomycotina includes organisms like the white rot fungi that are the only organisms capable of substantial lignin degradation (41); the Lingzhi mushroom (*Ganoderma lucidum*) used in the traditional Chinese medicine for more than 2000 years (42); tree root pathogens such as the major forest pathogen annosum root rot (*Heterobasidion annosum*) and *Armillaria bulbosa*, one of the largest and oldest of all organisms (43); mushrooms; and others. Pucciniomycotina and Ustilaginomycotina contain plant pathogens such as cereal rusts (*Puccinia graminis*) and corn smut (*Ustilago maydis*).

Prior work showed that antizyme frameshifting is reproducible in heterologous systems from *S. pombe* to mammalian cells (44) (although not in *S. cerevisiae* (45)). The FS stimulatory pseudoknot present in a subset of invertebrates was confirmed by testing the oyster antizyme mRNA in mammalian cells. Polyamine levels can be manipulated in a refined manner in mammalian cells (46). The present study examines the frameshift stimulators in a subset of fungal antizymes. Currently there is no homologous system available for testing the fungal antizyme mRNAs being investigated here. Because the polyamine levels cannot readily be manipulated so effectively in the closer related *S. pombe*, the experiments involving manipulation of the polyamine levels were performed in mammalian cells.

The ancient origin of frameshifting in the antizyme gene and its evolution are reflected in the large diversity of its *cis*-acting stimulatory elements. Exploration of antizyme sequences from the different branches provides insights to the means by which the evolution preserves the essential traits while it navigates through diverse possibilities.

## Experimental Procedures

### 

#### 

##### Sequence Assembly and Analysis

Sequences were obtained and processed as described previously (47). Alignments were generated using Clustal Omega (48). Nucleotide and amino acid logos were generated with WebLogo (49).

##### Plasmids

Oligonucleotides were synthesized at Integrated DNA Technologies (IDT). Primer sequences are listed in supplemental Table S1. A synthesized antizyme gene with the sequence from *C. cinerea* was purchased from Gen Script Corporation, where it was cloned in the vector pUC57 using an EcoRV cloning strategy. This sequence was used as template to generate the WT sequence, which in turn was used as template for generating the other clones. The amplicons were generated by standard one-step or two-step PCR (as indicated in supplemental Table S1). All amplicons were digested with BglII and XhoI and cloned into BglII/XhoI-digested vector (50, 51). The vector pDluc contains *Renilla* and firefly luciferase genes, separated by a short cloning site. Both luciferases are under the control of an upstream SV40 promoter. The antizyme cassettes were inserted between the two luciferase genes such that the upstream *Renilla* luciferase is in-frame with ORF1 of antizyme, whereas the downstream firefly luciferase is in-frame with ORF2. All constructs were transformed in *Escherichia coli* strain DH5-α and were verified by sequencing with the primer PD1550. The in-frame (IF) controls were generated by using a template in which the U of the ORF1 UGA stop codon was deleted. The antizyme construct from the alanine scan series wherein the glycine codon at position −4 was substituted with an alanine codon was compared with the ShortWT IF control instead. For testing antizyme frameshifting in *S. pombe*, antizyme cassettes were designed using primers that introduced the restriction sites for BstE II and KpnI at the 5′ and 3′ end of the amplicon, respectively. Following digestion, the antizyme cassettes were cloned into BstE II/KpnI-digested vector PIU-LAC between GST and *lacZ* (44).

##### Cell Culture and Transfection

Human HEK293T cells were maintained as monolayer cultures, grown in DMEM supplemented with 10% FBS, 1 mm l-glutamine, and antibiotics at 37 °C in an atmosphere of 5% CO_2_. For Dual-Luciferase assays, 4 × 10^6^ HEK293T cells were plated in 10-cm tissue culture dishes. After 24 h, the cells were detached with trypsin, suspended in fresh medium, and transfected in triplicate with Lipofectamine 2000 reagent (Invitrogen), using the 1-day protocol in which suspended cells are added directly to the DNA complexes in half-area 96-well plates. For each transfection the following was added to each well: 25 ng of plasmid DNA, 0.2 μl of Lipofectamine 2000 in 25 μl of OptiMem (Gibco). 4 × 10^4^ cells in 50 μl of DMEM were added to the transfecting DNA complexes in each well. Transfected cells were incubated at 37 C in 5% CO_2_ for 20 h and assayed using the Dual-Luciferase assay. For the polyamine manipulation protocol, cells were grown in the presence of 2.5 mm difluoromethylornithine (DFMO) for 5 days prior to transfection. After the incubation with DFMO, the cells were transfected using the protocol described above. 4 × 10^4^ cells in 25 μl of DMEM, grown in the presence of 2.5 mm DFMO, were added to the transfecting DNA complexes in each well. Transfected cells were incubated at 37 C in 5% CO_2_ for 24 h. After 24 h, 50 μl of fresh medium was added containing 1 mm aminoguanidine, 2.5 mm DFMO, and either no polyamines or polyamines to achieve final concentrations specified by experimental requirements. The cells were incubated for an additional 20 h before being assayed.

##### Dual-Luciferase Assay

To measure the frameshifting efficiency of antizyme mRNA cassettes, a Dual-Luciferase assay was employed. Relative light units were measured on a Veritas microplate luminometer fitted with two injectors (Turner Biosystems). The firefly and *Renilla* luciferase assay buffers were prepared as described (52). Transfected cells were washed once with 1× PBS and then lysed in 12.6 μl of 1× passive lysis buffer (Promega), and light emission was measured following injection of 50 μl of each luciferase substrate buffer. The product of the upstream *Renilla* luciferase gene reflects zero-frame translation. Synthesis of the downstream firefly luciferase is dependent on frameshifting when the ORF1 stop codon is in the ribosomal A-site. Therefore, for each data point firefly translation activity was normalized relative to the *Renilla* activity. The data were obtained from three independent transfection experiments each performed in triplicate. The nine data points for each construct were averaged, and standard deviations calculated. The frameshifting efficiency (calculated as a percentage) is obtained by comparing firefly to *Renilla* activity ratio of each antizyme cassette to its corresponding in-frame control cassette. Where statistical significance was calculated, an unpaired two sample *t* test with two tailed distribution was applied.

##### S. pombe Strains and Culture

*S. pombe* WT strain *h-s leu1-32 ura4 ade6-210* was used in these experiments. Plasmids were prepared from *E. coli* strain DH5-α and were transformed by a standard electroporation protocol. Edinburgh minimal medium, complete medium supplement, and complete medium supplement without uracil were all purchased from Sunrise Science Products (San Diego, CA).

##### Assay of β-Galactosidase Activity in S. pombe

The method used to assay the β-galactosidase activity in *S. pombe* was based on that described by Guarente (53). For each experiment, three single colonies of each construct were analyzed. The experiments were performed in two or three independent replicas. As for the Dual-Luciferase assay, the frameshift efficiencies were calculated as percentage by comparing the activities of the antizyme cassettes to the activities of their corresponding in-frame controls.

##### In Vitro Transcription

Capped and polyadenylated mRNAs were transcribed *in vitro* by T7 RNA polymerase (54) using DNA fragments generated from two rounds of PCR (see list of PCR primers). The yield of RNA was quantified using ImageQuantTL by comparison to known amounts of standard markers using ethidium bromide-stained agarose gels imaged with a GE Typhoon Trio instrument. Plasmid pPR301 was linearized with EcoRI to use as template for mRNA synthesis (54).

##### Primer Extension Inhibition (Toe Print) Assays

For toe-printing *Neurospora crassa* ribosomes in cell-free extracts that were synthesizing nascent peptides containing the partial antizyme domain, translation reaction mixtures (10 μl) supplemented with 0, 10, or 50 μm of spermidine were programmed with 60 ng of mRNA and incubated at 26 °C for 10 min. As controls, Luc mRNA containing wild type AAP uORF was translated in the presence of 10 μm or 2 mm Arg. Cycloheximide was added to translation reactions at a final concentration of 0.5 μg/μl before the incubation started or at the end of the incubation. Toe-print assays were performed as described (55). Toe-printing assay in rabbit reticulocyte lysate (RRL) was carried out as described (56). Briefly, *in vitro* transcribed mRNAs were incubated either in RRL supplemented with 15 mm MgCl_2_ to block both initiation and translation or in RRL-pretreated with cycloheximide, to block elongation but to allow 80S complex formation. Alternatively, RRL was treated with cycloheximide after 5 min of incubation with mRNA to block ongoing translation. Reverse transcription was then carried out with radioactively labeled oligonucleotide, and cDNA products were resolved on sequencing gel.

## Results

Intending to probe putative regulatory *cis*-acting sequences using a comparative genomics approach, we assembled sequences from additional Basidiomycota species (see “Experimental Procedures”). The current present set contains 55 such antizyme mRNA sequences (Fig. 2). One of the sequences was partial and was used only for the analysis of the 5′ conserved region.

### 

#### 

##### Comparative Sequence Analysis of the 5′ Conserved Region

Comparison of the 55 Basidiomycota sequences from the current set revealed that the 5′ conserved element was present in all 34 sequences from Agaricomycetes class, as well as in one sequence from class Dacrymycetes of Agaricomycotina. Antizyme sequences from the class Tremellomycetes of Agaricomycotina, as well as all the sequences of species outside of Agaricomycotina subphylum, did not show similarity at the amino acid level in the vicinity of the shift site and were excluded from further analysis of the 5′ sequence. Consequently the 35 antizyme sequences were employed for the current analysis of the 5′ conserved element (Fig. 2*A* and supplemental Sequence List S1). The sequence from *Dacryopinax sp*. class Dacrymycetes represents the maximum sequence divergence. Nucleotide and amino acid sequence alignments were used to generate respective sequence logos for ORF1 (Fig. 2*A*). All Agaricomycete antizyme mRNA sequences analyzed plus the sequence from Dacrymycetes have the frameshift site UUU-UGA.

Analysis of the 35 sequences confirms the previous observation that within the region 5′ adjacent to module A, there is high conservation at the peptide level (Fig. 2*A*) accompanied by synonymous substitutions in the corresponding codons at their third positions (note *downward pointing arrows* in the *third line* in Fig. 2*A*). The situation with module A itself is considered below. Additional conservation, previously unnoticed, is observed further upstream. Prominent among the other conserved features in ORF1 are two adjacent codons, encoding absolutely conserved Ala and Val, which show a high rate of synonymous substitutions. Another feature contained within the first half of ORF1 is a region of ∼50 nucleotides, 29 of which are absolutely conserved. This region appears conserved at the nucleotide level, and the most conserved nucleotides potentially form an RNA stem with 14 base-pairs and predicted stability of at least 30 kcal/mol (Fig. 2*A*).

##### Comparative Analysis of the 3′ RNA Putative Stimulatory Structure

The new analysis revealed that a 3′ structure that is homologous to the one identified in Agaricomycotina can be formed in a number of antizyme mRNA sequences belonging to two other subphyla of Basidiomycota. The broad distribution of this putative stimulator suggests its importance in many antizymes from Basidiomycota. 54 antizyme sequences having the first 72 nucleotides of ORF2 were aligned to show the conservation in the region encoding the putative secondary structure (Fig. 2, *B* and *C*, and supplemental Sequence List S2). Antizyme sequences from the class Tremellomycetes of Agaricomycotina (*e.g.* the “yeast-like” human pathogen *Cryptococcus neoformans*) did not seem to possess similar structure folding potential and were excluded from further analysis of the 3′ putative structure (Fig. 2*B*).

The 3′ structure in subphyla Ustilaginomycotina and Pucciniomycotina is similar to the one already published for Agaricomycotina (Fig. 2, *B* and *C*). There are extensive co-variant changes in stem 2. Stem 2, especially in antizyme mRNAs from Ustilaginomycotina and Pucciniomycotina, has several bulged nucleotides or nucleotide mismatches. No mismatches and very few co-variations are present in stem 1 from Agaricomycetes, but the sequences from non-Agaricomycotina species show numerous co-variations. In all the sequences but one, there is no break in base-pairing at the junction between stem 1 and stem 2, which is consistent with the previous suggestion that they may co-axially stack on each other.

All examined Basidiomycota sequences possessing the putative RNA structure contain the absolutely conserved four-nucleotide sequence AAAU abutting the 5′ end of stem 1. The corresponding region appears to be unstructured but might be part of unconventional ternary interactions.

The sequence of loop 1 is highly variable, both in composition and length. It can be as short as 3 nucleotides, as in *Piriformospora indica*, or as long as 28 nucleotides, as in *Fomitiporia mediterranea*. By contrast, the sequence of loop 2 is the most highly conserved region within the potential 3′ structure. Six of the seven nucleotides that comprise the loop are absolutely conserved. The loop has features, suggesting that it may exist in something other than a single-stranded state (9, 36) similar to previously published RNA triloop structures (57).

##### Experimental Analysis: Polyamine Levels and Degree of Relevance of Sequence Flanking the Putative Nascent Peptide Encoding Signal

Previously our lab developed a protocol for a fine manipulation of the polyamine levels in mammalian HEK293T cells (46). The lowest polyamine levels were achieved by pretreatment of HEK293T cells with DFMO, which is an irreversible inhibitor of ornithine decarboxylase (58). DFMO pretreatment, combined with adding polyamines to the media, allows controllable manipulation of free polyamine levels (46). Based on preliminary titration experiments with spermidine (SPD) (data not shown), three data points were chosen for most experiments presented here: DFMO treatment alone, DFMO plus 50 μm SPD, and DFMO plus 2 mm SPD.

**FIGURE 3.**
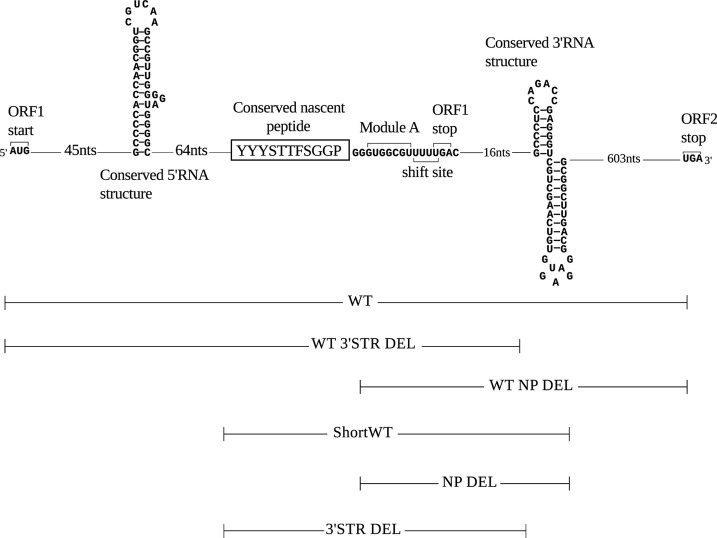
**Schematic representation of Agaricomycotina antizyme mRNA with conserved regions.** The sequence used in the scheme is that of *C. cinerea* antizyme mRNA. Shown from the *left* are the ORF1 start codon, a conserved putative RNA structure occurring 106 nucleotides 5′ of the shift site, the putative nascent peptide signal shown in a *box*, the module A sequence, shift site, and ORF1 stop codon. 19 nucleotides 3′ of the shift site, a conserved putative 3′ RNA structure was previously identified. *Below*, extent of the sequence present in six antizyme constructs used in this study.

**FIGURE 4.**
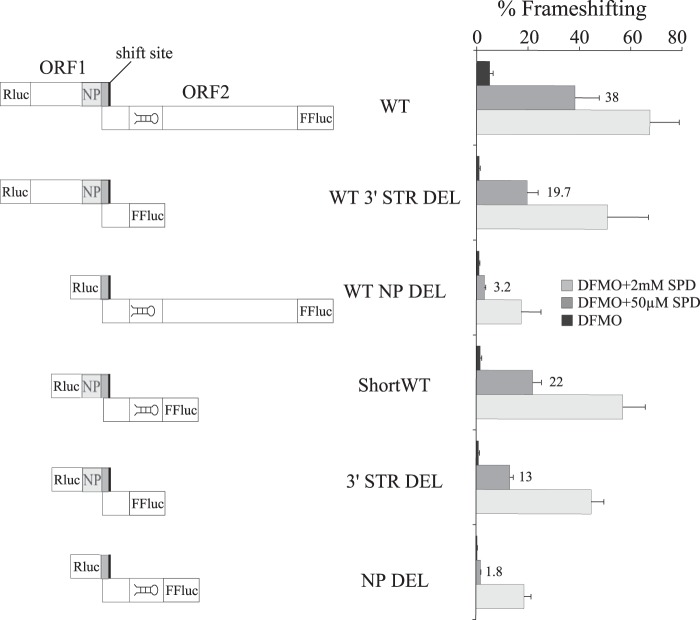
**Establishing the boundaries of the 5′ and 3′ stimulatory elements of *C. cinerea* antizyme mRNA from tests in HEK293T cells.** The WT construct contains the full-length ORF1 and ORF2 of the *C. cinerea* antizyme mRNA. The WT 3′ STR DEL construct contains full-length ORF1 but has a deletion starting 19 nucleotides 3′ of the shift site that includes the putative 3′ RNA structure. The WT NP DEL construct has a full-length ORF2, but the sequence encoding the conserved nascent peptide was deleted, leaving intact the sequence of module A and the shift site. The ShortWT construct contains the last 42 nucleotides of ORF1 including the sequence encoding the conserved nascent peptide, the sequence of module A, and the shift site, plus the first 64 nucleotides of ORF2 including the putative 3′ RNA structure. 3′ STR DEL construct is a derivative of ShortWT with the region encoding the putative 3′ RNA structure deleted. NP DEL construct is another derivative of ShortWT in which the sequence encoding the putative nascent peptide signal was deleted. All constructs were tested in three conditions: DFMO treatment (*black bars*), DFMO treatment plus 50 μm SPD (*gray bars*), and DFMO treatment plus 2 mm SPD (*light gray bars*).

We designed frameshift cassettes based on the sequence of *C. cinerea* antizyme mRNA that were cloned between *Renilla* and firefly luciferase with antizyme ORF1 in-frame with *Renilla* and firefly in-frame with antizyme ORF2 (Figs. 3 and 4). The WT cassette contained the full sequence of both ORFs. The antizyme sequence in a construct termed ShortWT starts at the 5′ end of the putative nascent peptide encoding sequence and extends to the 3′ end of the conserved RNA structure 3′ of the frameshift site (Figs. 3 and 4).

In the context of the WT cassette, deletion of sequence from the 3′ end of ORF2 up to that specifying the 5′ end of the structure 3′ of the shift site (WT 3′ STR DEL) yielded 19.7% frameshifting efficiency with DFMO plus 50 μm SPD treatment. This resulted in 1.9-fold reduction compared with WT levels (38%) (Fig. 4). Also in the context of the WT cassette, a deletion from the 5′ end of ORF1 to the 3′ nucleotides of the region encoding the putative nascent peptide signal (WT NP DEL) yielded 3.2% frameshifting efficiency with DFMO plus 50 μm SPD treatment. This is a 12-fold reduction compared with WT.

In the context of ShortWT, the corresponding deletion of the structure 3′ of the shift site (3′ STR DEL) or of the putative nascent peptide encoding sequence (NP DEL) were tested with DFMO plus 50 μm SPD treatment, yielding frameshifting levels of 13 and 1.8%, respectively. Compared with their control, in this case ShortWT, these levels involve the same reduction, 1.7- and 12-fold, as their counterparts in full-length WT context (Fig. 4). (The frameshifting efficiency of ShortWT is 22%.) The results suggest that the region specifying both the putative nascent peptide signal and the 3′ RNA structure has the same capacity to enhance frameshifting levels in the context of full-length WT and ShortWT.

However, the absolute frameshifting efficiency values of WT and its derivative constructs WT 3′ STR DEL and WT NP DEL were higher than that of ShortWT and its derivative constructs 3′ STR DEL and NP DEL, respectively. In addition, the fold difference was dependent on polyamine concentrations. When comparing WT and ShortWT FS efficiencies, with DFMO treatment alone a reduction of 3.1-fold was observed. Treatment with DFMO plus 50 μm SPD and DFMO plus 2 mm SPD resulted in a 1.7- and 1.2-fold reduction, respectively (Fig. 3, compare *WT* and *ShortWT*). The results suggest that there are additional stimulatory elements other than the regions encoding the putative nascent peptide signal and the conserved 3′ RNA structure, which are not present in ShortWT. A detailed exploration of proximal and distal stimulatory elements is outside the scope of the present study, which focuses mainly on the effect of the putative nascent peptide signal on frameshifting in the context of ShortWT.

**FIGURE 5.**
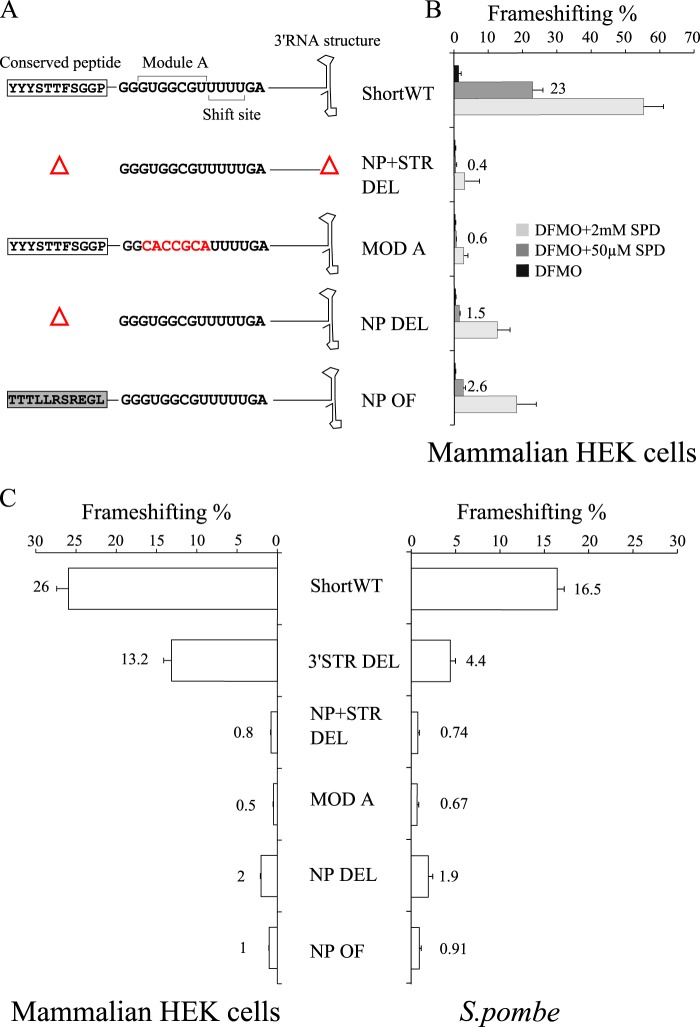
**Assessing the role of the conserved sequences on frameshifting from tests in HEK293T cells and *S. pombe*.** *A*, schematic representation of ShortWT and its derivative cassettes used in *B–D*. ShortWT, 3′ STR DEL, and NP DEL are as described in Fig. 4. The nucleotide sequences of module A and the shift site are indicated. The amino acid sequence of the putative nascent peptide signal (*box*) is shown instead of the nucleotide sequence encoding it. The NP+STR DEL construct has both sequences encoding the putative nascent peptide signal and the 3′ RNA structure deleted so that it contains only the 30 nucleotide sequence surrounding the frameshift site. The MOD A construct has the sequence of module A changed to its complement; the altered sequence is in *red*. The NP OF construct has the sequence coding for the nascent peptide put out of frame by deleting a nucleotide (U) at the 5′ end of the sequence and inserting a nucleotide (G) at its 3′ end. The altered nascent peptide sequence is *boxed* in *gray. B*, frameshift efficiencies of ShortWT and its derivative constructs tested in HEK293T cells treated with DFMO and supplemented with spermidine (*SPD*). The constructs were tested in three conditions: DFMO treatment only (*black bars*), DFMO treatment plus 50 μm SPD (*gray bars*), and DFMO treatment plus 2 mm SPD (*light gray bars*). *C*, frameshift efficiencies of ShortWT and its derivative constructs tested in HEK293T cells (*left*) and *S. pombe* (*right*) where the polyamine content was not manipulated.

One of the additional frameshift cassettes based on ShortWT had both regions encoding the putative nascent peptide signal and the 3′ RNA structure deleted but retained module A (5′ of the shift site), the shift site, and 19 nucleotides 3′ of the shift site (Fig. 5*A*, *NP*+*STR DEL*). This yielded 0.4% frameshifting with DFMO + 50 μm SPD treatment, which is a 57-fold reduction compared with ShortWT (Fig. 5*B*). With the same treatment, a derivative of ShortWT in which the sequence of module A was substituted with its complement yielded 0.6% frameshifting, a 38-fold reduction (Fig. 5, *A* and *B*, *MOD A*).

To obtain the frameshift efficiencies of the ShortWT and its derivatives in cells with endogenous polyamine concentrations, we tested the cassettes from Fig. 5*A*, as well as the 3′ STR DEL cassette from Fig. 4 in mammalian HEK293T cells where the polyamine levels were not manipulated (Fig. 5*C*, *left*). ShortWT yielded a frameshift efficiency of 26%, and its derivative with the 3′ RNA structure deleted yielded a frameshift efficiency of 13% (Fig. 5*C*, *3′ STR DEL*). NP+STR DEL and MOD A exhibited greatly reduced frameshift efficiencies: 0.8 and 0.5%, respectively (Fig. 5*C*).

The frameshifting levels with ShortWT and its derivatives tested in cells treated with DFMO + 50 μm SPD closely matched the frameshifting levels obtained in HEK293T cells with endogenous polyamine levels (Fig. 5, *B* and *C*). This observation suggested that the DFMO + 50 μm SPD treatment brings free intracellular polyamines close to their endogenous levels. In addition, DFMO + 50 μm SPD supported the greatest difference in frameshifting efficiency between ShortWT and its derivative constructs.

The mammalian HEK293T cell culture is a heterologous system for the analysis of regulatory frameshifting in the decoding of fungal antizyme mRNAs. The ShortWT and its derivative frameshift cassettes (Fig. 5*A*) were also fused to the +1 frame of *lacZ* and transfected in *S. pombe* cells. When tested in *S. pombe*, β-galactosidase assays extrapolate to 16.5% frameshifting with the ShortWT (Fig. 5*C*, *right*) (compared with 26% in HEK293T cells). Deletion of the 3′ RNA structure in 3′ STR DEL yielded 4.4% frameshifting efficiency, a 3.75-fold reduction compared with ShortWT. As with HEK293T cells, the two other constructs, NP+STR DEL and MOD A, exhibited dramatically reduced frameshifting efficiencies: double deletion of the putative nascent peptide encoding signal and the 3′ RNA structure produced a 22-fold reduction with frameshift levels of 0.74% (Fig. 5*C*, *NP*+*STR DEL*). In the ShortWT derivative, changing the module A sequence to its complement yielded a 25-fold reduction with frameshift levels of 0.67% (Fig. 5*C*, *MOD A*).

##### The Putative Nascent Peptide Signal

To probe the effect of the putative nascent peptide signal, the sequence encoding it was either deleted (NP DEL) or placed out of frame by deleting one nucleotide in the beginning of the sequence and inserting one nucleotide after it (NP OF) (Fig. 5*A*). Both alterations were done in the context of ShortWT, *i.e.* retaining the previously identified structure 3′ of the shift site. They were tested in mammalian cells and *S. pombe*. Corresponding great reductions, 9- and 13-fold for NP DEL and 18- and 26-fold for NP OF, in frameshift levels were observed in the two systems, respectively (Fig. 5*C*). A similar effect was observed in HEK293T cells with manipulated polyamine levels, with the greatest fold difference being obtained with DFMO + 50 μm SPD (Fig. 5*B*).

**FIGURE 6.**
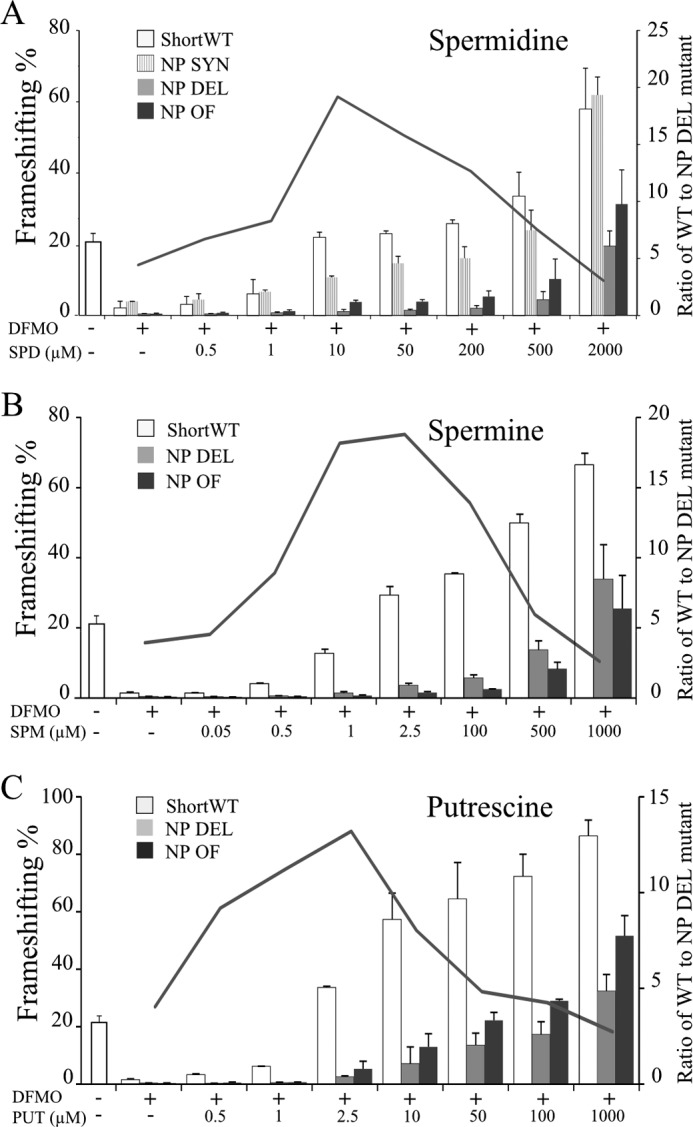
**Titration of polyamines with ShortWT and 5′ mutant cassettes.** ShortWT, NP DEL, and NP OF constructs are as in Figs. 4 and 5. The NP SYN construct introduces 11 synonymous changes in the sequence encoding the putative nascent peptide signal. The *leftmost white column* in each graph represents the frameshift level with ShortWT cassette in untreated cells; *i.e.* cells in which free polyamines are in homeostasis. The curve represents the ratio of ShortWT to NP DEL frameshifting across the titration gradient. *A*, titration of spermidine (*SPD*) with ShortWT (*white columns*), NP DEL (*gray columns*), NP OF (*black columns*), and NP SYN (*striped columns*). *B*, titration of spermine (*SPM*) with ShortWT (*white columns*), NP DEL (*gray columns*), and NP OF (*black columns*). *C*, titration of putrescine (*PUT*) with ShortWT (*white columns*), NP DEL (*gray columns*), and NP OF (*black columns*).

To further test whether the phylogenetically conserved 5′ stimulator within ShortWT functions at the amino acid or nucleotide levels, an additional cassette was generated. In this construct, NP SYN, the sequence of the encoded peptide was preserved, but the nucleotide sequence encoding it was altered at the third base of 11 codons by introducing synonymous substitutions. ShortWT, NP Del, NP OF, and NP SYN were then tested in a SPD titration experiment. Synonymous substitutions had little effect on frameshifting efficiencies compared with ShortWT through most SPD supplementation concentrations tested (Fig. 6*A*). Even in the SPD concentration range where there was some difference (10–500 μm), the frameshifting efficiencies of NP SYN were closer to ShortWT than to NP DEL or NP OF. Complete deletion of the sequence (NP DEL) and its out of frame variant (NP OF) resulted in similar greatly reduced frameshifting efficiencies throughout the concentration gradient. These results provide further evidence that high frameshifting efficiency depends on the peptide sequence (nascent peptide signal) rather than on its encoding nucleotide sequence. The greatest difference in FS efficiency between wild type or synonymous cassettes and those with altered peptide sequence was observed with intermediate SPD concentrations. This is illustrated by plotting the ratio of ShortWT to NP DEL frameshifting levels. This ratio was ∼5 at the lowest concentrations of spermidine and peaks at ∼19 with cells treated with DFMO and supplemented with 1 to 50 μm spermidine. It then declines at the highest concentrations of SPD (Fig. 6*A*). Interestingly, the position of the peak coincided with the range of spermidine supplementation that corresponds to endogenous levels of free polyamines in HEK293T cells.

Titration experiments with another main cellular polyamine, spermine, and with putrescine, were also performed. The peak of the ratio of ShortWT to NP DEL frameshifting levels with spermine was at DFMO plus 1–2.5 μm spermine, and with putrescine, it was at DFMO plus 2.5 μm putrescine (Fig. 6, *B* and *C*).

**FIGURE 7.**
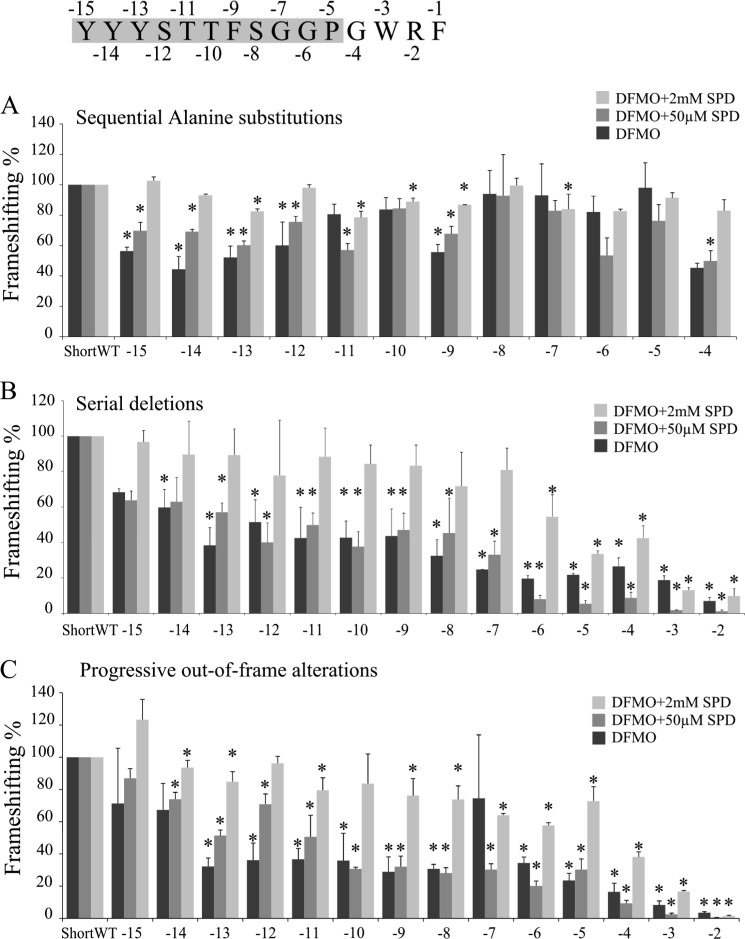
**Analysis of the 5′ nascent peptide stimulator.** The sequence of the 5′ nascent peptide signal (residues −15 to −5) is shown at the *top* of the figure and is highlighted in *gray*. The frameshifting efficiencies of three series of mutant constructs are presented as percentages of ShortWT efficiency. All constructs were tested in three conditions: DFMO treatment (*black bars*), DFMO treatment plus 50 μm SPD (*gray bars*), and DFMO treatment plus 2 mm SPD (*light gray bars*). Statistically significant results are marked by *asterisks. A*, sequential alanine substitutions of individual codons at positions from −15 to −4. *B*, serial deletions of codons from position −15 to −2. *C*, progressive out of frame alterations of codons at positions −15 to −2.

The contribution of individual amino acids of the nascent peptide to the stimulatory effect on frameshifting was assessed by testing three series of constructs. In an alanine scan series, each codon of the conserved peptide sequence (from position −15 to −5) was sequentially replaced with an alanine codon (each had an in-frame control). None of the individual alanine substitutions produced a dramatic effect on frameshifting efficiency, suggesting that amino acid identity at any one position is not crucial (Fig. 7*A*). The second, or deletion, series of constructs had a sequentially increasing number of codons from −15 to −2 deleted. In the third, or out of frame, series, increasing one-codon increments from −15 to −2 of the nascent peptide encoding sequence were put out of frame. This was accomplished by deleting one U nucleotide at the 5′ end of the sequence and adding one U nucleotide at the 3′ end of each increment. The results from the series of deletion and out of frame constructs are consistent with those from the alanine scan and indicate that the effect of residue deletion/alteration is cumulative, with progressive deletions yielding greater reductions of frameshifting efficiency (Fig. 7, *B* and *C*). Interestingly, the effect of the peptide alterations on the sensitivity of frameshifting to spermidine level is different with the different concentrations of SPD supplementation tested. At the highest concentration of SPD, the frameshift site preserved a near maximum efficacy when the first eight amino acid residues were altered. By contrast, under conditions mimicking endogenous polyamine levels (DFMO + 50 μm SPD) and under low polyamine concentration (DFMO only), frameshifting was significantly reduced with as few as the first three amino acid residues being altered (Fig. 7, *B* and *C*). Deletion or out of frame mutations that encroach on module A result in dramatic reduction of frameshifting efficiency, especially under condition of DFMO treatment plus 50 μm SPD supplementation.

##### Nascent Peptide Signal and Its Relationship to Module A

**FIGURE 8.**
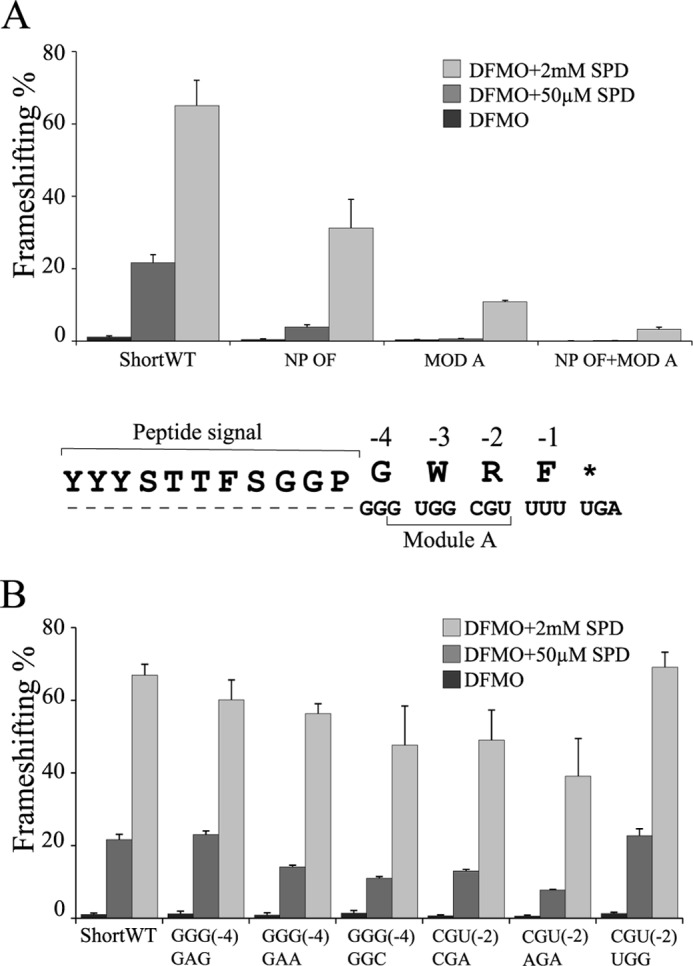
**Testing the effect of module A on antizyme frameshifting.** ShortWT, NP OF, and MOD A constructs are described in Figs. 4 and 5*A*. The constructs were tested in three conditions: DFMO treatment (*black bars*), DFMO treatment plus 50 μm SPD (*gray bars*), and DFMO treatment plus 2 mm SPD (*light gray bars*). *A*, NP+MOD A construct combines an out of frame nascent peptide encoding sequence and a module A sequence that is the complement of its WT. *B*, frameshifting efficiencies with ShortWT and its derivative cassettes with nucleotide substitutions in the codons at positions −4 or −2. The GGG(−4)GAG and GGG(−4)GAA constructs have the glycine codon (GGG) at position −4 changed to the glutamate (GAG) or (GAA) codon, either by one or two nucleotide substitutions, respectively. The GGG(−4)GGC cassette has the third G nucleotide of that codon changed to C. In the CGU(−2)CGA construct, the third nucleotide (U) in the CGU (Arg) codon at position −2 is changed to an A. CGU(−2)AGA has two nucleotide changes that, however, do not alter the encoded amino acid (Arg). In the CGU(−2)UGG construct, the CGU (Arg) codon at position −2 is changed to UGG (Trp) codon.

To elucidate the effect of module A on frameshifting stimulation, two constructs with altered module A sequence were tested. The peptide signal encoding sequence is unaltered in MOD A in which module A sequence was substituted with its complement. In construct NP OF+MOD A, in addition to the module A substitution, the sequence of codons −15 to −5 that encode the nascent peptide signal, is placed out of frame. When tested under the three SPD supplementation conditions, frameshifting efficiencies, with both NP OF+MOD A and MOD A constructs, were dramatically lower compared with those with ShortWT or NP OF (Fig. 8*A*). This suggests that the peptide sequence alone is insufficient for high levels of frameshifting or conversely that module A is essential for efficient frameshifting.

To investigate whether module A extends 7 nucleotides 5′ of the frameshift site, as previously suggested (36), we designed three constructs introducing nucleotide substitutions within the codon at position (−4) relative to the stop codon. The third nucleotide position in this codon, a G nucleotide, is the first nucleotide of module A, as proposed (36). In the GGG(−4)GGC cassette, the third nucleotide of this codon was changed from the wild type G to C, a substitution that preserved the identity of the encoded amino acid (Gly). The other two constructs tested at the same time have Glu at position (−4); however, in the GGG(−4)GAG construct, the Glu was encoded by a GAG codon, whereas in GGG(−4)GAA, it was encoded by a GAA codon. We chose to substitute the native (Gly) with (Glu), because in at least two mushroom antizyme mRNAs, the naturally occurring codon at position −4 is GAG Glu instead of GGG Gly. This amino acid change does not change the identity of the nucleotide at position 3. With SPD supplementation mimicking endogenous levels of polyamines, the construct GGG(−4)GAA yielded a frameshifting efficiency closer to that of the GGG(−4)GGC construct, whereas the construct GGG(−4)GAG yielded a frameshifting efficiency similar to that of ShortWT (Fig. 8*B*). The results suggest that the identity of the nucleotide at position 3 of codon −4 is more important than the identity of the amino acid encoded, consistent with it being part of the nucleotide specific module A. The nucleotides in the 5′ adjacent codon, UGG (Trp), cannot be changed without changing the amino acid identity at this position and so precluding this type of analysis.

The codon at position −2 was altered to test whether it functions at the nucleotide or amino acid levels. Cassettes CGU(−2)CGA and CGU(−2)AGA introduced one and two nucleotide substitutions, respectively, but both encode Arg, which is the wild type amino acid at that position (−2). Both constructs yielded reduced frameshifting levels with the one having two nucleotide substitutions exhibiting a greater reduction (Fig. 8*B*). The greatest effect was seen with DFMO + 50 μm SPD supplementation: frameshifting was reduced to 60% (CGU(−2)CGA) and 36% (CGU(−2)AGA) of ShortWT. We also tested the construct CGU(−2)UGG, which has two nucleotide substitutions that change the identity of the original Arg to Trp. This is a naturally occurring variation at codon (−2) in some Basidiomycotal antizyme mRNAs. Curiously, this alteration did not change the wild type FS levels.

**FIGURE 9.**
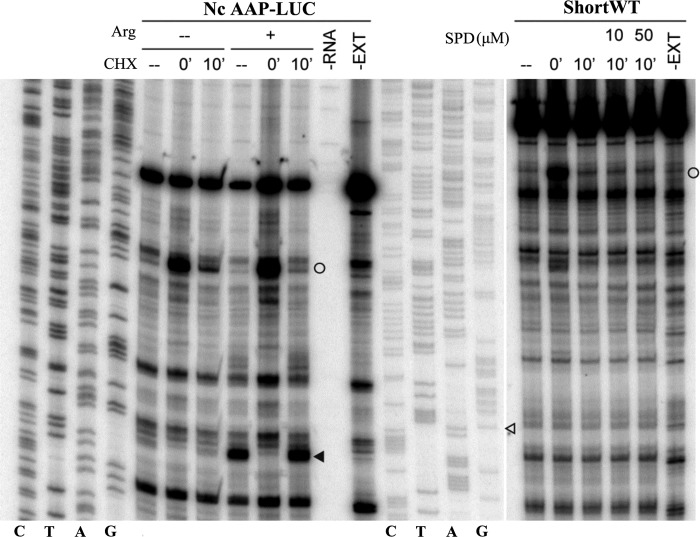
**Toe-printing of ribosomes translating *C. cinerea* antizyme mRNA in *N. crassa* extracts.** The mRNAs used to program translation in *N. crassa* extracts are indicated on the *top*. On the *left*, reaction mixtures contained 10 μm (−) or 2 mm (+) Arg and 10 μm each of the other 19 amino acids. On the right, reaction mixtures contained 10 μm of the 20 amino acids and 0, 10, or 50 μm of SPD. Cycloheximide was added to the reactions at the indicated time points. Lanes indicated as −*RNA* and −*EXT* show toe-printing of extract without added RNA and of RNA in the absence of extract, respectively. The same primer was also used for dideoxynucleotide sequencing of pRR301 (*left sequencing markers*) and pShortWT-IF (*right sequencing markers*). The *open circles* indicate the positions of the toe-prints corresponding to ribosomes with the initiation AUG codon in the P-site. The *black arrowhead* indicates the position of the toe-print corresponding to ribosomes with the AAP uORF termination codon in the A-site. The *open arrowhead* indicates the position of the G nucleotide in the ORF1 stop codon of ShortWT.

**FIGURE 10.**
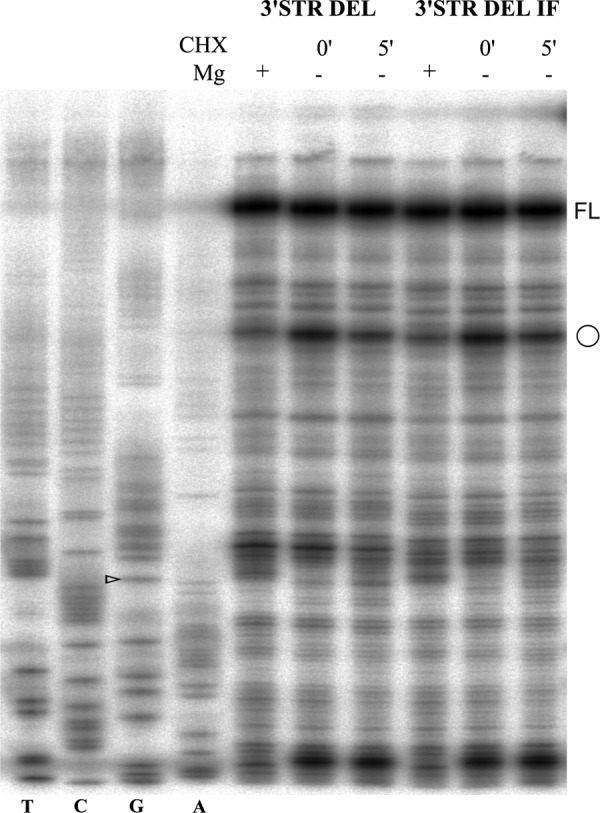
**Toe-printing of 3′ STR DEL and 3′ STR DEL IF mRNAs in RRL.** Translation conditions are labeled on the top of the gel. The *open circle* indicates the main initiation signal at the AUG. The position of the G nucleotide in the shift site is indicated by an *open arrowhead*.

Finally, we tested the potential of the stimulatory nascent peptide and module A in ShortWT context to cause a pause that is detectable using a toe-printing assay in *N. crassa* extracts (59). The positive control was the AAP encoding sequence in the presence or absence of externally added Arg (4) (Fig. 9). Stalling was not observed with the antizyme cassette under the conditions tested. Toe-printing with the 3′ STR DEL construct and its derivative with the U of the stop codon deleted in RRL gave a similar result (Fig. 10).

## Discussion

### 

#### 

##### Nascent Peptide

The results provide experimental and further phylogenetic evidence for a nascent peptide stimulator of Agaricomycetes antizyme mRNA +1 frameshifting. When the sequence encoding the 11-amino acid peptide (encoded −15 to −5 codons 5′ of the shift site) is placed out of frame, frameshifting is reduced similarly to that of a complete deletion. In contrast, compared with wild type, introducing 11 synonymous nucleotide changes in the same region had a small effect on frameshifting. Serial 5′ deletions, out of frame mutations, and an alanine scan (Fig. 7) suggest that the contribution of the amino acids to the effect is cooperative with no single amino acid being crucial for the stimulatory effect.

In contrast to the nascent peptide signal effects in *S. cerevisiae*, the peptide signal described here acts within the ribosome mediating the frameshift event. The 11-amino acid-long peptide signal is expected to occupy approximately one-third of the peptide exit tunnel. It is longer than MAGDIS, the hexapeptide product of the inhibitory uORF in mammalian AdoMetDC mRNA (60), but substantially shorter than the nascent peptide signal responsible for StopGo/Stop-Carry on (61). Atomic level structure analysis of *S. cerevisiae* ribosomes reveals a constriction in the exit tunnel (62), that is, the counterpart of that in bacteria at which SecM works; in bacteria, this is approximately one-third of the way into the tunnel from the PTC. Because of positioning of the Agaricomycete nascent peptide signal beginning 3 amino acids distant from the peptidyl transferase center (PTC) at the time of frameshifting, it may span the constriction likely also present in its ribosomes.

Small molecules, other than polyamines, can influence nascent peptide effects more generally within the peptide exit tunnel, and an increasing number of examples are known where a specific nascent sequence in the exit tunnel causes a ribosome stall in eukaryotes (63, 64) and in bacteria (65, 66). In some cases (67), the ribosome stalling occurs just prior to, or during, the decoding of a stop codon in the ribosomal A site, such that termination is inhibited. Because frameshifting and other recoding events are in competition with standard decoding, inhibition of the standard decoding step should push the equilibrium toward the recoding event (68).

The results from the toe-printing assay could reflect instability of the particular stalled complex involved, even though the counterpart paused complex in the positive control was stable. However, it is more likely that at the sensitivity of the assay, no detectable pausing occurs. The peptide effect might not be due to ribosome stalling. If so, it would be conceptually similar to one case in bacteria recently identified where ketolides promote −1 frameshifting when translating the leader peptide that results in activation of the translation of the downstream gene (6). Recently highly sensitive single-molecule fluorescence has been applied to study the dynamics of a 5′ stimulatory element on bacterial −1 frameshifting (69). As FRET approaches are developed for counterpart studies in eukaryotes, much more refined step time data should be forthcoming. Knowledge of the likely noncanonical ribosome states in antizyme frameshifting are likely from such studies and other approaches, especially cryo-electron microscopy.

The results presented here suggest that the role of the Agaricomycetes nascent peptide stimulators, presumably via interaction with exit tunnel components, is not simply to increase the efficiency of frameshifting but also to contribute to making the process especially sensitive to intracellular free polyamines at their endogenous levels. This is a novel feature for the function of *cis*-acting sequences in antizyme mRNA frameshifting.

##### Polyamine Level

Previous analyses of *cis*-acting stimulators of frameshifting in antizyme genes were usually performed in a way that only examined their effect in two extreme conditions with regard to polyamine concentrations: cells depleted for polyamines and supplemented with the highest dose of polyamines. This approach fails to investigate what function the *cis*-acting stimulators have in intermediate concentrations of polyamines closer to their endogenous levels. When a spermidine titration experiment was performed with a wild type construct and three mutant constructs affecting the nascent peptide stimulator, a significant result was observed. The mutant constructs that substituted the amino acid sequence of this region had greatly reduced frameshifting efficiencies as compared with wild type and synonymous cassettes. This effect was especially pronounced at intermediate polyamine concentrations, which stimulated the wild type and the synonymous mutant cassette, far more than the two mutants that change the amino acid sequence of the nascent peptide (Fig. 6*A*). The point on the titration experiment where the difference in stimulation was greatest coincides with the DFMO + polyamine supplementation that induces frameshifting levels in ShortWT close to 22%. This is very close to the frameshifting observed with the same sequence in untreated cells. In other words, the difference in response to polyamines between mutant cassettes that do or do not encode altered peptide sequence is greatest under conditions that most closely mimic endogenous levels of free polyamines. This suggests that the sensor function of the nascent peptide stimulator is physiologically relevant. The potential for differential affinity sites for polyamine binding with contrasting effects on frameshifting makes it likely that discerning the mechanism will not be a simple task.

##### Module A

One of the 5′ stimulators of antizyme frameshifting, apparently present in at least most antizyme homologs other than antizyme 3 (39, 70), is a sequence of six to seven nucleotides immediately abutting the shift site. At the time of the frameshifting, the 5′ adjacent codon to the shift site UUU_U would be in the E-site or in transit. Prior studies with different types of frameshifting have shown the importance of E-site codon interactions in some cases (71–73). In Agaricomycete antizyme mRNA, the E-site codon position with the greatest variability is definitely the third position (Fig. 2*A*). Experimentally changing the CGU (Arg) to CGA (Arg) or AGA (Arg) reduced frameshifting at all polyamine concentrations tested, with two changes leading to more severe reduction (Fig. 8*B*). In some Basidiomycotal antizyme mRNAs, outside of the Agaricomycetes lineage, there is a natural variation at the same codon, −2, that replaces the CGU Arg with UGG Trp. Substituting CGU with UGG, involving the same two codon positions did not reduce frameshifting at any of the polyamine concentrations tested (Fig. 8*B*). This is consistent with the overall strength of E-site codon:anticodon, irrespective of individual nucleotide position, being relevant but is a weak inference.

Changing codon −4 from GGG (Gly) to GAG (Glu) had no effect on the efficiency, but changing it to GAA (Glu) resulted in a 40% reduction and to GCG (Ala) had a 50% reduction. These data raise the possibility that the encoded amino acid may have an effect in the peptide exit tunnel, and the nucleotide sequence may have an effect with the mRNA exit tunnel, perhaps even in interactions with rRNA.

Mutating module A to its complementary sequence had a similar effect on polyamine stimulation across the titration gradient as seen with the nascent peptide mutants. Although the number of data points was limited (Fig. 8*A*), this suggests that module A too could be involved in sensitizing the shift site to polyamines in their endogenous level range. As introduced above, module A is widely evolutionarily conserved, and its original likely predates that of the nascent peptide signal in Agaricomycetes, which has a restricted range. Although module A and the nascent peptide signal work at the same time, it may well be that future work will reveal instances of programmed frameshifting where the only 5′/5′ encoded stimulator is a nascent peptide signal. The involvement of nascent peptide signals in stop codon readthrough, a different class of recoding, is also to be expected.

##### Perspective

The experiments examining the mode of function of the conserved elements in Agaricomycetes antizyme mRNAs revealed that in addition to their stimulatory effect on the frameshift efficiency, these elements seem to sensitize the frameshift site for polyamines, especially in the range of their endogenous concentrations. This model provides an example for how the *cis*-acting elements in antizyme mRNAs could exert their function.

## Author Contributions

J. F. A. and M. M. Y. with the aid of one of those acknowledged designed the study. M. M. Y. performed the experiments shown in Figs. 3–8. C. W. and M. S. S. performed and analyzed the experiments shown in Fig. 9. D. E. A. performed the experiments shown in Fig. 10. All authors reviewed the results and approved the final version of the manuscript written by M. M. Y. and J. F. A.

## Supplementary Material

Supplemental Data

## Footnotes

3 I. P. Ivanov and J. F. Atkins, unpublished results.

## Footnotes

2 The abbreviations used are:

uORF: upstream open reading frame
AAP: arginine attenuator peptide
IF: in-frame
DFMO: d,l-α-difluoromethylornithine
RRL: rabbit reticulocyte lysate
SPD: spermidine
FS: frameshifting.

## References

[1] Nissen P. Hansen J. Ban N. Moore P. B. Steitz T. A. (2000) The structural basis of ribosome activity in peptide bond synthesis. Science 289, 920–930 10937990 10.1126/science.289.5481.920

[2] Wilson D. N. Beckmann R. (2011) The ribosomal tunnel as a functional environment for nascent polypeptide folding and translational stalling. Curr. Opin. Struct. Biol. 21, 274–282 21316217 10.1016/j.sbi.2011.01.007

[3] Ruan H. Shantz L. M. Pegg A. E. Morris D. R. (1996) The upstream open reading frame of the mRNA encoding *S*-adenosylmethionine decarboxylase is a polyamine-responsive translational control element. J. Biol. Chem. 271, 29576–29582 8939886 10.1074/jbc.271.47.29576

[4] Wang Z. Sachs M. S. (1997) Ribosome stalling is responsible for arginine-specific translational attenuation in *Neurospora crassa*. Mol. Cell. Biol. 17, 4904–4913 9271370 10.1128/mcb.17.9.4904 PMC232343

[5] Arenz S. Ramu H. Gupta P. Berninghausen O. Beckmann R. Vázquez-Laslop N. Mankin A. S. Wilson D. N. (2014) Molecular basis for erythromycin-dependent ribosome stalling during translation of the ErmBL leader peptide. Nat. Commun. 5, 3501 24662426 10.1038/ncomms4501 PMC4133097

[6] Gupta P. Kannan K. Mankin A. S. Vázquez-Laslop N. (2013) Regulation of gene expression by macrolide-induced ribosomal frameshifting. Mol. Cell 52, 629–642 24239289 10.1016/j.molcel.2013.10.013 PMC3874817

[7] Hayashi S. Murakami Y. Matsufuji S. (1996) Ornithine decarboxylase antizyme: a novel type of regulatory protein. Trends Biochem. Sci. 21, 27–30 8848835

[8] Kahana C. (2009) Regulation of cellular polyamine levels and cellular proliferation by antizyme and antizyme inhibitor. Essays Biochem. 46, 47–61 20095969 10.1042/bse0460004

[9] Ivanov I. P. Matsufuji S. (2010) Autoregulatory frameshifting in antizyme gene expression governs polyamine levels from yeast to mammals. In Recoding: Expansion of Decoding Rules Enriches Gene Expression (Atkins J. F. Gesteland R. F., eds) Springer, New York

[10] Sakata K. Kashiwagi K. Igarashi K. (2000) Properties of a polyamine transporter regulated by antizyme. Biochem. J. 347, 297–303 10727431 PMC1220960

[11] Pegg A. E. Casero R. A. Jr. (2011) Current status of the polyamine research field. Methods Mol. Biol. 720, 3–35 21318864 10.1007/978-1-61779-034-8_1 PMC3652263

[12] Gutierrez E. Shin B. S. Woolstenhulme C. J. Kim J. R. Saini P. Buskirk A. R. Dever T. E. (2013) eIF5A promotes translation of polyproline motifs. Mol. Cell 51, 35–45 23727016 10.1016/j.molcel.2013.04.021 PMC3744875

[13] Lightfoot H. L. Hall J. (2014) Endogenous polyamine function: the RNA perspective. Nucleic Acids Res. 42, 11275–11290 25232095 10.1093/nar/gku837 PMC4191411

[14] Auvinen M. Paasinen A. Andersson L. C. Hölttä E. (1992) Ornithine decarboxylase activity is critical for cell transformation. Nature 360, 355–358 1280331 10.1038/360355a0

[15] Poulin R. Coward J. K. Lakanen J. R. Pegg A. E. (1993) Enhancement of the spermidine uptake system and lethal effects of spermidine overaccumulation in ornithine decarboxylase-overproducing L1210 cells under hyposmotic stress. J. Biol. Chem. 268, 4690–4698 8444843

[16] Seiler N. Raul F. (2005) Polyamines and apoptosis. J. Cell. Mol. Med. 9, 623–642 16202210 10.1111/j.1582-4934.2005.tb00493.x PMC6741638

[17] Kramer D. L. Chang B. D. Chen Y. Diegelman P. Alm K. Black A. R. Roninson I. B. Porter C. W. (2001) Polyamine depletion in human melanoma cells leads to G_1_ arrest associated with induction of p21WAF1/CIP1/SDI1, changes in the expression of p21-regulated genes, and a senescence-like phenotype. Cancer Res. 61, 7754–7762 11691789

[18] Ray R. M. Bhattacharya S. Bavaria M. N. Viar M. J. Johnson L. R. (2014) Antizyme (AZ) regulates intestinal cell growth independent of polyamines. Amino acids 46, 2231–2239 24930035 10.1007/s00726-014-1777-0 PMC4134379

[19] Ivanov I. P. Firth A. E. Atkins J. F. (2010) Recurrent emergence of catalytically inactive ornithine decarboxylase homologous forms that likely have regulatory function. J. Mol. Evol. 70, 289–302 20217058 10.1007/s00239-010-9331-5

[20] Murakami Y. Ohkido M. Takizawa H. Murai N. Matsufuji S. (2014) Multiple forms of mouse antizyme inhibitor 1 mRNA differentially regulated by polyamines. Amino acids 46, 575–583 24077669 10.1007/s00726-013-1598-6

[21] Fujita K. Murakami Y. Hayashi S. (1982) A macromolecular inhibitor of the antizyme to ornithine decarboxylase. Biochem. J. 204, 647–652 7126159 10.1042/bj2040647 PMC1158403

[22] Bunjobpol W. Dulloo I. Igarashi K. Concin N. Matsuo K. Sabapathy K. (2014) Suppression of acetylpolyamine oxidase by selected AP-1 members regulates DNp73 abundance: mechanistic insights for overcoming DNp73-mediated resistance to chemotherapeutic drugs. Cell Death Differ. 21, 1240–1249 24722210 10.1038/cdd.2014.41 PMC4085530

[23] Matsufuji S. Matsufuji T. Miyazaki Y. Murakami Y. Atkins J. F. Gesteland R. F. Hayashi S. (1995) Autoregulatory frameshifting in decoding mammalian ornithine decarboxylase antizyme. Cell 80, 51–60 7813017 10.1016/0092-8674(95)90450-6 PMC7133313

[24] Namy O. Galopier A. Martini C. Matsufuji S. Fabret C. Rousset J. P. (2008) Epigenetic control of polyamines by the prion [PSI+]. Nat. Cell Biol. 10, 1069–1075 19160487 10.1038/ncb1766

[25] Ray R. M. Viar M. J. Johnson L. R. (2012) Amino acids regulate expression of antizyme-1 to modulate ornithine decarboxylase activity. J. Biol. Chem. 287, 3674–3690 22157018 10.1074/jbc.M111.232561 PMC3281678

[26] Atkins J. F. Lewis J. B. Anderson C. W. Gesteland R. F. (1975) Enhanced differential synthesis of proteins in a mammalian cell-free system by addition of polyamines. J. Biol. Chem. 250, 5688–5695 167021

[27] Tabor C. W. Tabor H. (1984) Polyamines. Annu. Rev. Biochem. 53, 749–790 6206782 10.1146/annurev.bi.53.070184.003533

[28] Kurian L. Palanimurugan R. Gödderz D. Dohmen R. J. (2011) Polyamine sensing by nascent ornithine decarboxylase antizyme stimulates decoding of its mRNA. Nature 477, 490–494 21900894 10.1038/nature10393

[29] Rato C. Amirova S. R. Bates D. G. Stansfield I. Wallace H. M. (2011) Translational recoding as a feedback controller: systems approaches reveal polyamine-specific effects on the antizyme ribosomal frameshift. Nucleic Acids Res. 39, 4587–4597 21303766 10.1093/nar/gkq1349 PMC3113565

[30] Balasundaram D. Dinman J. D. Tabor C. W. Tabor H. (1994) SPE1 and SPE2: two essential genes in the biosynthesis of polyamines that modulate +1 ribosomal frameshifting in *Saccharomyces cerevisiae*. J. Bacteriol. 176, 7126–7128 7961484 10.1128/jb.176.22.7126-7128.1994 PMC197094

[31] Ivanov I. P. Matsufuji S. Murakami Y. Gesteland R. F. Atkins J. F. (2000) Conservation of polyamine regulation by translational frameshifting from yeast to mammals. EMBO J. 19, 1907–1917 10775274 10.1093/emboj/19.8.1907 PMC302018

[32] Ivanov I. P. Anderson C. B. Gesteland R. F. Atkins J. F. (2004) Identification of a new antizyme mRNA +1 frameshifting stimulatory pseudoknot in a subset of diverse invertebrates and its apparent absence in intermediate species. J. Mol. Biol. 339, 495–504 15147837 10.1016/j.jmb.2004.03.082 PMC7125782

[33] Weiss R. B. Dunn D. M. Dahlberg A. E. Atkins J. F. Gesteland R. F. (1988) Reading frame switch caused by base-pair formation between the 3′ end of 16S rRNA and the mRNA during elongation of protein synthesis in *Escherichia coli*. EMBO J. 7, 1503–1507 2457498 10.1002/j.1460-2075.1988.tb02969.x PMC458402

[34] Larsen B. Wills N. M. Gesteland R. F. Atkins J. F. (1994) rRNA-mRNA base pairing stimulates a programmed −1 ribosomal frameshift. J. Bacteriol. 176, 6842–6851 7961443 10.1128/jb.176.22.6842-6851.1994 PMC197052

[35] Gurvich O. L. Näsvall S. J. Baranov P. V. Björk G. R. Atkins J. F. (2011) Two groups of phenylalanine biosynthetic operon leader peptides genes: a high level of apparently incidental frameshifting in decoding *Escherichia coli* pheL. Nucleic Acids Res. 39, 3079–3092 21177642 10.1093/nar/gkq1272 PMC3082878

[36] Ivanov I. P. Atkins J. F. (2007) Ribosomal frameshifting in decoding antizyme mRNAs from yeast and protists to humans: close to 300 cases reveal remarkable diversity despite underlying conservation. Nucleic Acids Res. 35, 1842–1858 17332016 10.1093/nar/gkm035 PMC1874602

[37] Loughran G. Firth A. E. Atkins J. F. (2011) Ribosomal frameshifting into an overlapping gene in the 2B-encoding region of the cardiovirus genome. Proc. Natl. Acad. Sci. U.S.A. 108, E1111–E1119 22025686 10.1073/pnas.1102932108 PMC3219106

[38] Li Y. Treffers E. E. Napthine S. Tas A. Zhu L. Sun Z. Bell S. Mark B. L. van Veelen P. A. van Hemert M. J. Firth A. E. Brierley I. Snijder E. J. Fang Y. (2014) Transactivation of programmed ribosomal frameshifting by a viral protein. Proc. Natl. Acad. Sci. U.S.A. 111, E2172–E2181 24825891 10.1073/pnas.1321930111 PMC4040542

[39] Ivanov I. P. Rohrwasser A. Terreros D. A. Gesteland R. F. Atkins J. F. (2000) Discovery of a spermatogenesis stage-specific ornithine decarboxylase antizyme: antizyme 3. Proc. Natl. Acad. Sci. U.S.A. 97, 4808–4813 10781085 10.1073/pnas.070055897 PMC18314

[40] Ivanov I. P. Gesteland R. F. Atkins J. F. (1998) A second mammalian antizyme: conservation of programmed ribosomal frameshifting. Genomics 52, 119–129 9782076 10.1006/geno.1998.5434

[41] Floudas D. Binder M. Riley R. Barry K. Blanchette R. A. Henrissat B. Martinez A. T. Otillar R. Spatafora J. W. Yadav J. S. Aerts A. Benoit I. Boyd A. Carlson A. Copeland A. Coutinho P. M. de Vries R. P. Ferreira P. Findley K. Foster B. Gaskell J. Glotzer D. Górecki P. Heitman J. Hesse C. Hori C. Igarashi K. Jurgens J. A. Kallen N. Kersten P. Kohler A. Kües U. Kumar T. K. Kuo A. LaButti K. Larrondo L. F. Lindquist E. Ling A. Lombard V. Lucas S. Lundell T. Martin R. McLaughlin D. J. Morgenstern I. Morin E. Murat C. Nagy L. G. Nolan M. Ohm R. A. Patyshakuliyeva A. Rokas A. Ruiz-Duenas F. J. Sabat G. Salamov A. Samejima M. Schmutz J. Slot J. C. St John F. Stenlid J. Sun H. Sun S. Syed K. Tsang A. Wiebenga A. Young D. Pisabarro A. Eastwood D. C. Martin F. Cullen D. Grigoriev I. V. Hibbett D. S. (2012) The Paleozoic origin of enzymatic lignin decomposition reconstructed from 31 fungal genomes. Science 336, 1715–1719 22745431 10.1126/science.1221748

[42] Chen S. Xu J. Liu C. Zhu Y. Nelson D. R. Zhou S. Li C. Wang L. Guo X. Sun Y. Luo H. Li Y. Song J. Henrissat B. Levasseur A. Qian J. Li J. Luo X. Shi L. He L. Xiang L. Xu X. Niu Y. Li Q. Han M. V. Yan H. Zhang J. Chen H. Lv A. Wang Z. Liu M. Schwartz D. C. Sun C. (2012) Genome sequence of the model medicinal mushroom *Ganoderma lucidum*. Nat. Commun. 3, 913 22735441 10.1038/ncomms1923 PMC3621433

[43] Anderson J. B. Catona S. (2014) Genomewide mutation dynamic within a long-lived individual of *Armillaria gallica*. Mycologia 106, 642–648 24891414 10.3852/13-367

[44] Ivanov I. P. Gesteland R. F. Matsufuji S. Atkins J. F. (1998) Programmed frameshifting in the synthesis of mammalian antizyme is +1 in mammals, predominantly +1 in fission yeast, but −2 in budding yeast. RNA 4, 1230–1238 9769097 10.1017/s1355838298980864 PMC1369695

[45] Matsufuji S. Matsufuji T. Wills N. M. Gesteland R. F. Atkins J. F. (1996) Reading two bases twice: mammalian antizyme frameshifting in yeast. EMBO J. 15, 1360–1370 8635469 PMC450040

[46] Howard M. T. Shirts B. H. Zhou J. Carlson C. L. Matsufuji S. Gesteland R. F. Weeks R. S. Atkins J. F. (2001) Cell culture analysis of the regulatory frameshift event required for the expression of mammalian antizymes. Genes Cells 6, 931–941 11733031 10.1046/j.1365-2443.2001.00477.x

[47] Ivanov I. P. Loughran G. Atkins J. F. (2008) uORFs with unusual translational start codons autoregulate expression of eukaryotic ornithine decarboxylase homologs. Proc. Natl. Acad. Sci. U.S.A. 105, 10079–10084 18626014 10.1073/pnas.0801590105 PMC2481320

[48] Sievers F. Higgins D. G. (2014) Clustal Omega, accurate alignment of very large numbers of sequences. Methods Mol. Biol. 1079, 105–116 24170397 10.1007/978-1-62703-646-7_6

[49] Crooks G. E. Hon G. Chandonia J. M. Brenner S. E. (2004) WebLogo: a sequence logo generator. Genome Res. 14, 1188–1190 15173120 10.1101/gr.849004 PMC419797

[50] Grentzmann G. Ingram J. A. Kelly P. J. Gesteland R. F. Atkins J. F. (1998) A dual-luciferase reporter system for studying recoding signals. RNA 4, 479–486 9630253 PMC1369633

[51] Fixsen S. M. Howard M. T. (2010) Processive selenocysteine incorporation during synthesis of eukaryotic selenoproteins. J. Mol. Biol. 399, 385–396 20417644 10.1016/j.jmb.2010.04.033 PMC2916059

[52] Dyer B. W. Ferrer F. A. Klinedinst D. K. Rodriguez R. (2000) A noncommercial Dual-Luciferase enzyme assay system for reporter gene analysis. Anal. Biochem. 282, 158–161 10860516 10.1006/abio.2000.4605

[53] Guarente L. (1983) Yeast promoters and lacZ fusions designed to study expression of cloned genes in yeast. Methods Enzymol. 101, 181–191 6310321 10.1016/0076-6879(83)01013-7

[54] Wu C. Amrani N. Jacobson A. Sachs M. S. (2007) The use of fungal *in vitro* systems for studying translational regulation. Methods Enzymol. 429, 203–225 17913625 10.1016/S0076-6879(07)29010-X

[55] Wu C. Wei J. Lin P. J. Tu L. Deutsch C. Johnson A. E. Sachs M. S. (2012) Arginine changes the conformation of the arginine attenuator peptide relative to the ribosome tunnel. J. Mol. Biol. 416, 518–533 22244852 10.1016/j.jmb.2011.12.064 PMC3568992

[56] Dmitriev S. E. Pisarev A. V. Rubtsova M. P. Dunaevsky Y. E. Shatsky I. N. (2003) Conversion of 48S translation preinitiation complexes into 80S initiation complexes as revealed by toeprinting. FEBS Lett. 533, 99–104 12505166 10.1016/s0014-5793(02)03776-6

[57] Mitrasinovic P. M. (2006) On the structural features of hairpin triloops in rRNA: from nucleotide to global conformational change upon ligand binding. J. Struct. Biol. 153, 207–222 16439157 10.1016/j.jsb.2005.12.001

[58] Pegg A. E. (2006) Regulation of ornithine decarboxylase. J. Biol. Chem. 281, 14529–14532 16459331 10.1074/jbc.R500031200

[59] Wang Z. Sachs M. S. (1997) Arginine-specific regulation mediated by the *Neurospora crassa* arg-2 upstream open reading frame in a homologous, cell-free in vitro translation system. J. Biol. Chem. 272, 255–261 8995256 10.1074/jbc.272.1.255

[60] Mize G. J. Ruan H. Low J. J. Morris D. R. (1998) The inhibitory upstream open reading frame from mammalian S-adenosylmethionine decarboxylase mRNA has a strict sequence specificity in critical positions. J. Biol. Chem. 273, 32500–32505 9829983 10.1074/jbc.273.49.32500

[61] Donnelly M. L. Luke G. Mehrotra A. Li X. Hughes L. E. Gani D. Ryan M. D. (2001) Analysis of the aphthovirus 2A/2B polyprotein “cleavage” mechanism indicates not a proteolytic reaction, but a novel translational effect: a putative ribosomal “skip.” J. Gen. Virol. 82, 1013–1025 11297676 10.1099/0022-1317-82-5-1013

[62] Yusupova G. Yusupov M. (2014) High-resolution structure of the eukaryotic 80S ribosome. Annu. Rev. Biochem. 83, 467–486 24580643 10.1146/annurev-biochem-060713-035445

[63] Luo Z. Freitag M. Sachs M. S. (1995) Translational regulation in response to changes in amino acid availability in *Neurospora crassa*. Mol. Cell. Biol. 15, 5235–5245 7565672 10.1128/mcb.15.10.5235 PMC230771

[64] Wei J. Wu C. Sachs M. S. (2012) The arginine attenuator peptide interferes with the ribosome peptidyl transferase center. Mol. Cell. Biol. 32, 2396–2406 22508989 10.1128/MCB.00136-12 PMC3434499

[65] Vázquez-Laslop N. Ramu H. Klepacki D. Kannan K. Mankin A. S. (2010) The key function of a conserved and modified rRNA residue in the ribosomal response to the nascent peptide. EMBO J. 29, 3108–3117 20676057 10.1038/emboj.2010.180 PMC2944061

[66] Gong F. Yanofsky C. (2002) Instruction of translating ribosome by nascent peptide. Science 297, 1864–1867 12228716 10.1126/science.1073997

[67] Cao J. Geballe A. P. (1996) Inhibition of nascent-peptide release at translation termination. Mol. Cell. Biol. 16, 7109–7114 8943366 10.1128/mcb.16.12.7109 PMC231714

[68] Atkins J. F. Gesteland R. F. (eds) (2010) Recoding: Expansion of Decoding Rules Enriches Gene Expression, Springer, New York

[69] Chen J. Petrov A. Johansson M. Tsai A. O'Leary S. E. Puglisi J. D. (2014) Dynamic pathways of −1 translational frameshifting. Nature 512, 328–332 24919156 10.1038/nature13428 PMC4472451

[70] Tosaka Y. Tanaka H. Yano Y. Masai K. Nozaki M. Yomogida K. Otani S. Nojima H. Nishimune Y. (2000) Identification and characterization of testis specific ornithine decarboxylase antizyme (OAZ-t) gene: expression in haploid germ cells and polyamine-induced frameshifting. Genes to cells 5, 265–276 10792465 10.1046/j.1365-2443.2000.00324.x

[71] Sanders C. L. Curran J. F. (2007) Genetic analysis of the E site during RF2 programmed frameshifting. RNA 13, 1483–1491 17660276 10.1261/rna.638707 PMC1950767

[72] Baranov P. V. Gesteland R. F. Atkins J. F. (2002) Release factor 2 frameshifting sites in different bacteria. EMBO Reports 3, 373–377 11897659 10.1093/embo-reports/kvf065 PMC1084053

[73] Léger M. Dulude D. Steinberg S. V. Brakier-Gingras L. (2007) The three transfer RNAs occupying the A, P and E sites on the ribosome are involved in viral programmed −1 ribosomal frameshift. Nucleic Acids Res. 35, 5581–5592 17704133 10.1093/nar/gkm578 PMC2018615

