# Supplementary material for: A Nascent Peptide Signal Responsive to Endogenous Levels of Polyamines Acts to Stimulate Regulatory Frameshifting on Antizyme mRNA
Source: J Biol Chem. 2015 May 21;290(29):17863–78. doi: 10.1074/jbc.M115.647065 (PMC4505036; doi:10.1074/jbc.M115.647065)
Supplement: Supplemental Data [file supp_290_29_17863__index.html]

A Nascent Peptide Signal Responsive to Endogenous Levels of Polyamines Acts to Stimulate Regulatory Frameshifting on Antizyme mRNA — A Nascent Peptide Signal Responsive to Endogenous Levels of Polyamines Acts to Stimulate Regulatory Frameshifting on Antizyme mRNA — Nascent Peptide Stimulator of Frameshifting — Supplemental Data 

# A Nascent Peptide Signal Responsive to Endogenous Levels of Polyamines Acts to Stimulate Regulatory Frameshifting on Antizyme mRNA

## Supplemental Data

- Supplemental Table 1 (.pdf, 234 KB) - Supplemental Table 1
- List of sequences 1 (.txt, 8 KB) - Basidiomycota antizyme mRNA sequences used for the analysis of ORF1
- List of sequences 2 (.txt, 41 KB) - Basidiomycota antizyme mRNA sequences used for the analysis of ORF2
