# Supplementary material for: A Nascent Peptide Signal Responsive to Endogenous Levels of Polyamines Acts to Stimulate Regulatory Frameshifting on Antizyme mRNA
Source: J Biol Chem. 2015 May 21;290(29):17863–78. doi: 10.1074/jbc.M115.647065 (PMC4505036; doi:10.1074/jbc.M115.647065)
Supplement: Supplemental Data [file supp_M115.647065_jbc.M115.647065-1.pdf]

A nascent peptide signal responsive to endogenous levels of polyamines acts to stimulate regulatory frameshifting on antizyme mRNA

Martina M. Yordanova[1], Cheng Wu[2], Dmitry E. Andreev[3], Matthew S. Sachs[2], John F. Atkins[1,4]

[1]School of Biochemistry and Cell Biology, University College Cork, Ireland.

[2]Department of Biology, Texas A & M University, College Station, TX77843-3258.

[3]Belozersky Institute of Physico-Chemical Biology, Lomonosov Moscow State University, Moscow, Russia.

[4]Department of Human Genetics, University of Utah, UT84112-5330, USA

SUPPLEMENTAL INFORMATION

Suplemental table 1 A. List of primers used in this study. **B**. Scheme for the generation of antizyme constructs through one or two step PCR.

Suplemental text file 1 List of the sequences used for analysis of ORF1 of the antizyme mRNAs from the Agaricomycotina fungal branch.

Suplemental text file 2 List of the sequences used for analysis of ORF2 of the antizyme mRNAs belonging to the Basidiomycota phylum.

Supplemental table 1. (*A*) List of the primers used for generating the antizyme constructs tested in this study.

1	GTCCCTCGAGTTACTACTACTCTACTACGTTCTCGG
2	GTCCAGATCTCGCCGAACTGCCTCTCACAC
3	GTCCAGATCTGCATTTTGCTTTGGGGGTGTC
4	TACTCTACTACGTTCTCGGGAGGGCCTGGCACCGCATTTTGACACCCCCAA
5	GTCCCTCGAGTACTACTACTCTACTACGTTCTCGGGAGGGCCTGGTCAC
6	CGTTCTCGGGAGGGCCTGGTCACCGCATTTTGACACCCCCAAAGC
7	CGTTCTCGGGAGGGCCTGGTCACCGCATTTGACACCCCCAAAGC
8	GTCCCTCGAGTTACTACTACTCTACTACGTTCTCGGGAGGG
9	ACGTTCTCGGGAGGGCCTGGGTGGTGGTTTTGACACCCC
10	GTCCCTCGAGTTACTACTACTCTACTACGTTCTCGGGAGGGCCT
11	ACGTTCTCGGGAGGGCCTGGGTGGTGGCCCTGACACCCCCAAA
12	GTCCCTCGAGTTACTACTACTCTACTACGTTCTCGGGAGGG
13	ACGTTCTCGGGAGGGCCTGGGGCGTGGTTTTGACACCCCC
14	GTCCCTCGAGTTACTACTACTCTACTACGTTCTCGGGAGGG
15	ACGTTCTCGGGAGGGCCTGGGGCGGCTTTTTGACACCCCC
16	ACGACTTTTTCAGGGGGACCAGGGTGGCGTTTTTGACACCC
17	GTCCCTCGAGTTATTATTATTCCACGACTTTTTCAGG
18	GTCCCTCGAGTTACTACTACTCTACTACGTTCTCGGGAGGGCCTGAGTGGCGTTTT
19	GTCCCTCGAGTTACTACTACTCTACTACGTTCTCGGGAGGGCCTGAATGGCGTTTT
20	GTCCCTCGAGTTACTACTACTCTACTACGTTCTCGGGAGGGCCTGGCTGGCGTTTTTGA
21	GGTAAGTACATCAAGAGCTTCGTG
22	CGAGTGGGTAGAATGGCGCTG
23	CTAAGCTTTGGGGGTGTCAAAACGCCA
24	TTAATGGCGTTTTGACACCCCCAAAGC
25	GTCCCTCGAGTTACTACTACTCTACTACGTTCTCGGGAGGGCCTGGGTGGCGATTTTGA
26	GTCCCTCGAGTTACTACTACTCTACTACGTTCTCGGGAGGGCCTGGGTGGAGATTTTGA
27	TCTACTACGTTCTCGGGAGGGCCTGGGTGGTGGTTTGACACCCC
28	TCTACTACGTTCTCGGGAGGGCCTGGGTGGTGGCCCGACACCCCCAAA
29	TCTACTACGTTCTCGGGAGGGCCTGGGGCGTGGTTTGACACCCCC
30	TCTACTACGTTCTCGGGAGGGCCTGGGGCGGCTTTTGACACCCCC
31	TCTACTACGTTCTCGGGAGGGCCTGAGTGGCGTTT
32	TCTACTACGTTCTCGGGAGGGCCTGAATGGCGTTT
33	TCTACTACGTTCTCGGGAGGGCCTGGCTGGCGTTTT
34	TCTACTACGTTCTCGGGAGGGCCTGGGTGGCGATTTGACACCCC
35	TCTACTACGTTCTCGGGAGGGCCTGGGTGGAGATTTGACACCCC
36	GTCCCTCGAGTTGGCGTTTTGACACCCCCAAAG
37	GTCCCTCGAGTGCCTACTACTCTACTACGTTCTCGGG

38	GTCCCTCGAGTTACGCCTACTCTACTACGTTCTCGGGAG
39	GTCCCTCGAGTTACTACGCCTCTACTACGTTCTCGGGAGG
40	GTCCCTCGAGTTACTACTACGCTACTACGTTCTCGGGAGGG
41	GTCCCTCGAGTTACTACTACTCTGCTACGTTCTCGGGAGGGC
42	GTCCCTCGAGTTACTACTACTCTACTGCCTTCTCGGGAGGGCCTG
43	GTCCCTCGAGTTACTACTACTCTACTACGGCCTCGGGAGGGCCTGG
44	GTCCCTCGAGTTACTACTACTCTACTACGTTCGCCGGAGGGCCTGGGTG
45	GTCCCTCGAGTTACTACTACTCTACTACGTTCTCGGCAGGGCCTGGGTGG
46	GTCCCTCGAGTTACTACTACTCTACTACGTTCTCGGGAGCCCCTGGGTGGCGTT
47	GTCCCTCGAGTTACTACTACTCTACTACGTTCTCGGGAGGGGCTGGGTGGCGTTT
48	GTCCCTCGAGTTACTACTACTCTACTACGTTCTCGGGAGGGCCTGCCTGGCGTTTTTGAC
49	GTCCCTCGAGTTACTACTCTACTACGTTCTCGGG
50	GTCCCTCGAGTTACTCTACTACGTTCTCGGGAG
51	GTCCCTCGAGTTCTACTACGTTCTCGGGAGGG
52	GTCCCTCGAGTACTACGTTCTCGGGAGGGC
53	GTCCCTCGAGTACGTTCTCGGGAGGGCCT
54	GTCCCTCGAGTTTCTCGGGAGGGCCTGGG
55	GTCCCTCGAGTTCGGGAGGGCCTGGGTG
56	GTCCCTCGAGTGGAGGGCCTGGGTGGC
57	GTCCCTCGAGTGGGCCTGGGTGGCGTTTT
58	GTCCCTCGAGTCCTGGGTGGCGTTTTTGACA
59	GTCCCTCGAGTGGGTGGCGTTTTTGACACCC
60	GTCCCTCGAGTTGGCGTTTTTGACACCCCCAAAG
61	GTCCCTCGAGTCGTTTTTGACACCCCCAA
62	GTCCCTCGAGTTTTTGACACCCCCAAAGCAAAATG
63	GTCCCTCGAGTACTTACTACTCTACTACGTTCTCGGG
64	GTCCCTCGAGTACTACTTACTCTACTACGTTCTCGGGAG
65	GTCCCTCGAGTACTACTACTTCTACTACGTTCTCGGGAGGGC
66	GTCCCTCGAGTACTACTACTCTTACTACGTTCTCGGGAGGGC
67	GTCCCTCGAGTACTACTACTCTACTTACGTTCTCGGGAGGGCCT
68	GTCCCTCGAGTACTACTACTCTACTACGTTTCTCGGGAGGGCCTGG
69	GTCCCTCGAGTACTACTACTCTACTACGTTCTTCGGGAGGGCCTGGGTG
70	GTCCCTCGAGTACTACTACTCTACTACGTTCTCGTGGAGGGCCTGGGTGGC
71	GTCCCTCGAGTACTACTACTCTACTACGTTCTCGGGATGGGCCTGGGTGGCGTT
72	GTCCCTCGAGTACTACTACTCTACTACGTTCTCGGGAGGGTCCTGGGTGGCGTTTT
73	GTCCCTCGAGTACTACTACTCTACTACGTTCTCGGGAGGGCCTTGGGTGGCGTTTTTGA
74	GTCCCTCGAGTACTACTACTCTACTACGTTCTCGG
75	CTACTCTACTACGTTCTCGGGAGGCCCTGGCGTGGCGTTTTTGACACC
76	GTCCCTCGAGTACTACTACTCTACTACGTTCTCGGGAGGGCCTGGGTGGTCGTTTTTGA
77	GTCCCTCGAGTACTACTACTCTACTACGTTCTCGGGAGGGCCTGGGTGGCGTTTTTTGA
78	TCGGGAGGGCCTGGGTGGCGTTTTTTGACACCCCCAAAGCAAAATGCC
79	GTCCCTCGAGTCGTTTTGACACCCCCAAAG
80	GTCCCTCGAGTGGGTGGCGTTTTGACACCC
81	GTCCCTCGAGTCCTGGGTGGCGTTTTGACA

82	GTCCCTCGAGTTTTGACACCCCCAAAGCAAAATG
83	CTCGAGTACTACTACTCTACTACGTTCTCGGGAGGGCCTGGGTGGTCGTTTTGA
84	GTCCCTCGAGTACTACTACTCTACTACGTTC
85	GTCCCTCGAGTACTACTACTCTACTACGTTCTCGGGAGGGCCTGGGTTGGCGTT
86	TACTCTACTACGTTCTCGGGAGGGCCTGGCACCGCATTTGACACCCCCAA
87	GTCCCTCGAGTGATGTGCTAGCGGTTTGCCATC
88	GTCCCTCGAGTGATGTGCTAGGAGGCTGCCATCTTCAGG
PD1550	CCGTCTTCGAGTGGGTAGAA
419	CAACAACAACAACAACgtgaTGAAGAACGAGCAGTAtTTCTAca
421	CCGACTCTAGAATTATTACACGG
418	CGCCGtaatacgactcactataGGGCAACAACAACAACAACAACgtga
421	CCGACTCTAGAATTATTACACGG
ZW4	TCCAGGAACCAGGGCGTA

(B) Generation of antizyme constructs through one or two step PCR.

**Construct**	**PCR 1**	**PCR 2**	**IF construct**	**PCR 1**	**PCR 2**
ShortWT	1+2		ShortWT IF	1+2	
WT	21+22		WT	21+22	
WT 3'STR DEL	21+3		WT 3'STR DEL IF	21,3	
WT NP DEL	60+22		WT NP DEL IF	60,22	
3'STR DEL	1+3		3'STR DEL IF	1+3	
NP DEL	60+2		NP DEL IF	60+2	
NP+STR DEL	60+3		NP+STR DEL IF	60+3	
NP SYN	16+2	17+2	NP SYN IF	16+2	17+2
MOD A	4+2	1+2	MOD A IF	86+2	1+2
NP OF+MOD A	6+2	5+2	NP OF+MOD A IF	7+2	5+2
Ala (-15)	37+2		Ala (-15) IF	37,2	
Ala (-14)	38+2		Ala (-14) IF	38+2	
Ala (-13)	39+2		Ala (-13) IF	39+2	
Ala (-12)	40+2		Ala (-12) IF	40+2	
Ala (-11)	41+2		Ala (-11) IF	41+2	
Ala (-10)	42+2		Ala (-10) IF	42+2	
Ala (-9)	43+2		Ala (-9) IF	43+2	
Ala (-8)	44+2		Ala (-8) IF	44+2	
Ala (-7)	45+2		Ala (-7) IF	45+2	
Ala (-6)	46+2		Ala (-6) IF	46+2	
Ala (-5)	47+2		Ala (-5) IF	47+2	
Ala (-4)	48+2				
Del (-15)	49+2		Del (-15) IF	49+2	
Del (-14)	50+2		Del (-14) IF	50+2	

Del (-13)	51+2		Del (-13) IF	51+2	
Del (-12)	52+2		Del (-12) IF	52+2	
Del (-11)	53+2		Del (-11) IF	53+2	
Del (-10)	54+2		Del (-10) IF	54+2	
Del (-9)	55+2		Del (-9) IF	55+2	
Del (-8)	56+2		Del (-8) IF	56+2	
Del (-7)	57+2		Del (-7) IF	57+2	
Del (-6)	58+2		Del (-6) IF	81+2	
Del (-5)	59+2		Del (-5) IF	80+2	
Del (-4)	60+2		Del (-4) IF	36+2	
Del (-3)	61+2		Del (-3) IF	79+2	
Del (-2)	62+2		Del (-2) IF	82+2	
OF (-15)	63+2		OF (-15) IF	63+2	
OF (-14)	64+2		OF (-14) IF	64+2	
OF (-13)	65+2		OF (-13) IF	65+2	
OF (-12)	66+2		OF (-12) IF	66+2	
OF (-11)	67+2		OF (-11) IF	67+2	
OF (-10)	68+2		OF (-10) IF	68+2	
OF (-9)	69+2		OF (-9) IF	69+2	
OF (-8)	70+2		OF (-8) IF	70+2	
OF (-7)	71+2		OF (-7) IF	71+2	
OF (-6)	72+2		OF (-6) IF	72+2	
OF (-5)	73+2		OF (-5) IF	x	
OF (-4)	75+2	74+2	OF (-4) IF	85+2	
OF (-3)	76+2		OF (-3) IF	83+2	
OF (-2)	78+2	77+2	OF (-2) IF	84+2	
GGG(-4)GAG	18+2		GGG(-4)GAG IF	31+2	1+2
GGG(-4)GAA	19+2		GGG(-4)GAA IF	32+2	1+2
GGG(-4)GGC	20+2		GGG(-4)GGC IF	33+2	1+2
CGU(-2)CGA	25+2		CGU(-2)CGA IF	34+2	1+2
CGU(-2)AGA	26+2		CGU(-2)AGA IF	35+2	1+2
CGU(-2)UGG	8+9+2		CGU(-2)UGG IF	27+2	1+2
